# Mapping the Substrate Specificity Landscape of PAD2 and PAD4 Enzymes

**DOI:** 10.1002/cbic.70408

**Published:** 2026-06-07

**Authors:** Adina Borbély, Arnold Steckel, Dávid Papp, Domonkos Pál, Katalin Uray, Viktória Goldschmidt Gőz, Gitta Schlosser

**Affiliations:** ^1^ MTA‐ELTE Lendület (Momentum) Ion Mobility Mass Spectrometry Research Group Department of Analytical Chemistry Institute of Chemistry ELTE Eötvös Loránd University Budapest Hungary; ^2^ Hevesy György PhD School of Chemistry Faculty of Science Institute of Chemistry ELTE Eötvös Loránd University Budapest Hungary; ^3^ MTA‐TTK Lendület (Momentum) Glycan Biomarker Research Group HUN‐REN Research Centre for Natural Sciences Budapest Hungary; ^4^ Doctoral School Semmelweis University Budapest Hungary; ^5^ Institute of Chemistry HUN‐REN–ELTE Research Group of Peptide Chemistry, ELTE Eötvös Loránd University Budapest Hungary

**Keywords:** citrullination, combinatorial library, mass spectrometry, PAD2, PAD4

## Abstract

Pathological citrullination of proteins is implicated in several diseases, including rheumatoid arthritis, neurodegenerative disorders, and cancer. This irreversible post‐translational modification is driven by the peptidyl arginine deiminase (PAD) enzyme family. Although the substrate specificity of the key PAD2 and PAD4 isotypes has been studied, the detailed role of individual amino acids from a multidimensional perspective remains unexplored. Here, we designed a combinatorial library of 256 synthetic peptides to comprehensively map PAD2 and PAD4 substrate preferences. Sixteen natural amino acids were examined in positions flanking the target arginine residue using combinatorial peptide mixtures produced by a split‐and‐mix synthetic strategy. Relative citrullination efficiencies following PAD treatment were analyzed using mass spectrometry, enhanced by cyclic ion mobility separation. PAD4 displayed higher substrate selectivity than PAD2. PAD2 efficiently citrullinated 233 of the 256 peptides, reaching ≥92% efficiency, with Glu and Pro in the *C*‐terminal position of the target arginine being unfavorable. In contrast, PAD4 showed lower overall conversion, with Pro strongly inhibiting citrullination at the C‐terminal position and multiple residues negatively affecting the N‐terminal position. Notably, Asn at either flanking position enhanced citrullination for both PAD enzymes. These findings highlighted the need to differentiate arginine citrullination from asparagine deamidation for accurate identification.

## Graphical abstract

Protein citrullination, driven by peptidyl arginine deiminases (PAD2, PAD4), is linked to multiple diseases. Using a combinatorial peptide library and advanced mass spectrometry, we reveal distinct substrate selectivity profiles of PAD2 and PAD4. While PAD2 broadly citrullinates arginine‐containing peptides, PAD4 is highly sequence‐restricted, with flanking residues critically shaping modification efficiency.

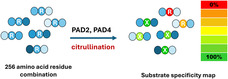

## 1 Introduction

**FIGURE 1:**
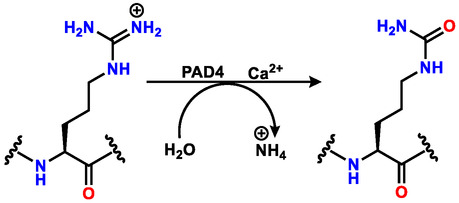
PAD4‐mediated arginine deimination.

Protein citrullination (deimination) is an important post‐translational modification (PTM) of arginine residues catalyzed by the peptidyl arginine deiminase (PAD) enzyme family. This irreversible conversion of peptidyl arginine to peptidyl citrulline though resulting in only a small mass increase of 0.984 Da, fundamentally alters protein chemistry by hydrolyzing a positively charged guanidino group into a neutral ureido group (Figure 1) [1]. This seemingly subtle shift has profound consequences, disrupting hydrogen bonding and driving significant conformational changes in protein structure, which, in turn, modulate the protein's function, activity, stability, and interactions with other proteins [2, 3, 4]. Dysregulated PAD activity, leading to elevated levels of citrullinated proteins, has been linked to a range of diseases, including rheumatoid arthritis (RA) [5], multiple sclerosis [6], Alzheimer's disease (AD) [7], and cancer [8].

Citrullination is regulated by five PAD isozymes (PAD1−4 and PAD6), which, despite their high sequence homology [4], exhibit distinct tissue localization and substrate specificity. PAD1 is predominantly expressed in the epidermis and in the male and female reproductive systems, while PAD3 is localized to hair follicles and keratinocytes. PAD2 and PAD4, on the other hand, have widespread distributions and play diverse roles. PAD2 is abundant in skeletal muscle, the central nervous system, inflammatory cells (neutrophils, macrophages, mast cells), secretory glands, and various cancer types. PAD4, previously also referred to as PAD5, is found in inflammatory cells (macrophages, neutrophils, eosinophils), granulocytes, and multiple cancer. PAD4 uniquely contains a nuclear localization signal, which allows it to penetrate the nucleus and catalyze the citrullination of histone proteins [9]. PAD6 is specifically expressed in early embryos and oocytes [10, 11, 12]. PAD activity is heavily calcium‐dependent [13, 14], but other factors—such as pH, bicarbonate or reducing agents (DTT, GHS, or thioredoxin)—also serve as critical coactivators [15, 16, 17].

In recent years, several immunological and mass spectrometric techniques have been developed to detect and quantify protein citrullination [18, 19]. While immunodetection techniques can be highly sensitive for identifying citrullinated proteins, and anti‐citrullinated protein antibodies serve as crucial pathological markers [20], these approaches cannot pinpoint the exact site of deimination. Mass spectrometry (MS), by contrast, is a powerful tool for site‐specific characterization of citrullination at the molecular level, thanks to its exceptional sensitivity and specificity [21]. However, reliably identifying citrullination remains technically challenging due to several complicating factors. Citrullinated proteins or peptides are typically present at substoichiometric levels, making precursor ion isolation not optimal, likely reducing the quality of MS/MS spectra [22]. Additionally, the deimination of arginine residues can be easily mistaken due to a more frequent PTM, the deamidation of Asn/Gln residues, which results in an identical mass shift of 0.9840 Da [23]. This overlap complicates the accurate identification, localization, and quantification of citrullination often leading to a high rate of false positives in proteome analyses [24]. To overcome these challenges, standard MS approaches must be strengthened with rigorous criteria to ensure the comprehensive and accurate identification of modified peptides, proteins, or proteomes. One widely used diagnostic marker is the characteristic neutral loss of HNCO [25] from citrullinated peptide precursors and fragment ions in CID/HCD spectra. Our group has also explored the “citrulline effect,” which refers to preferential cleavage at the C‐termini of citrulline residues [26, 27, 28]. In parallel, various strategies have been developed to improve specific steps of the MS‐based proteomic workflow, including fine‐tuning fragmentation for enhanced database search reliability [29], adopting data‐independent acquisition methods [30], and leveraging statistical [24, 31], and machine learning prediction models [32]. Achieving unambiguous identification of citrullination sites on the protein level is essential not only for determining the amino acid sequence adjacent to the modified residues, but also for revealing potential substrate preferences among the PAD enzyme family.

Despite the identification of several endogenous protein substrates for PAD enzymes and their increasing relevance to human disease, the distinct substrate specificities of PAD isotypes remain only partially understood. Early studies have uncovered both shared and PAD2‐ or PAD4‐specific citrullination sites in limited number of endogenous protein substrates, revealing that PAD2 generally catalyzes citrullination more efficiently, while PAD4 demonstrates greater regioselectivity [14, 33, 34, 35]. It has become clear that PAD enzyme activity is strongly influenced by the structure and sequence of peptide and protein substrates surrounding the target arginine [36, 37, 38, 39]. Detailed studies have primarily focused on exploring the substrate motif preferences of PAD2 and PAD4 through the analysis of synthetic peptides or by mapping citrullination in complex proteome digests. The single amino acid substitution approach, applied to known peptide substrates, aims to classify residues adjacent to the target arginine as either favorable or unfavorable for PAD2/PAD4‐catalyzed citrullination. Citrullination efficiencies of synthetic peptide libraries have been assessed relative to native peptide sequences, with preferred and disfavored residues defined in comparison to the original residue present near the central arginine [40, 41]. While these studies offer valuable insights into PAD2 and PAD4 substrate specificities, they also present conflicting results regarding the classification of favorable/unfavorable residues and are limited by challenges in comparative evaluation. Additionally, proteomic dataset‐based motif analysis has highlighted preferred amino acids in close proximity to the citrullination site [32], yet this model may be biased by the under‐ or overrepresentation of specific peptides or amino acid motifs within the data.

We present the first systematic investigation of PAD2 and PAD4 substrate specificity using a combinatorial peptide library. By analyzing the deimination activity of both enzymes on synthetic peptide substrates, we definitively pinpointed the influence of individual amino acids flanking the citrullination site. This allowed us to deliver a comprehensive, multidimensional assessment of specific recognition motifs, providing unprecedented clarity on their substrate preferences.

## 2 Results and Discussion

### 2.1 Design and Synthesis of the Peptide Library

To systematically explore the individual effects of all amino acids surrounding the arginine residue targeted by PAD enzymes, we designed, synthesized, and tested a unique combinatorial peptide library. This library was based on the general 9‐mer sequence H‐ASAZ_1_RZ_2_ASA‐NH_2_, where Z_1_ (position −1) and Z_2_ (position +1) represent all natural amino acids (except Arg, Cys, Met, and Trp) directly preceding and following the central arginine. Cys, Met, and Trp were excluded due to their high susceptibility to oxidation during peptide synthesis and subsequent sample handling. The first and last three amino acids (Ala‐Ser‐Ala) were chosen to minimize any influence on the citrullination efficiency of the central Arg, as these positions typically have little or no effect on the reaction [41]. In total, 256 peptides were synthesized using the split‐mix method of the combinatorial solid‐phase peptide synthesis to assess how neighboring amino acid side chains affect citrullination efficiency. The peptides were divided into 12 groups (Rmix1–Rmix12, sequences listed in Tables S1–S12), in a manner such that peptide isomers and isobars were separated into different groups, facilitating their distinction during MS analysis. Arginine‐containing peptides were incubated with human recombinant PAD2 and PAD4, and their citrullination efficiency was determined through mass spectrometric analysis by comparing enzyme‐treated samples to untreated controls. Comprehensive identification and characterization of citrullinated peptides were carried out by ion mobility separation combined with tandem MS.

### 2.2 Identification of Citrullinated Peptides by LC‐HDMS^**E**^

**FIGURE 2:**
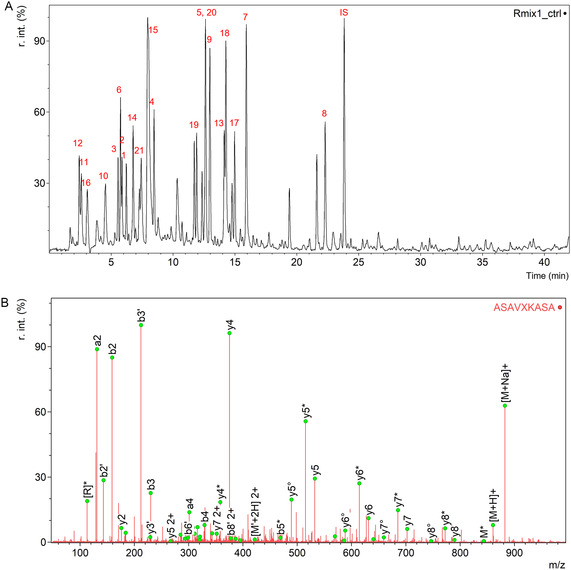
(A) Total ion chromatogram of the 21 oligopeptides from the first peptide mixture (Rmix1). “IS” refers to the internal standard peptide H‐ASAAVLASA‐NH_2_. Unlabeled peaks correspond to synthetic side products. (B) The MS^E^ spectrum of the H‐ASAVXKASA‐NH_2_ peptide. X = citrulline amino acid. Neutral losses are indicated as follows: ‘ = H_2_O, * = NH_3_, and ° = HNCO.

High‐definition MS^E^ (HDMS^E^) incorporating single‐pass cyclic ion mobility separation into both low and elevated collision energy scans was employed in our workflow, which enables isomeric or isobaric peptide ions to be discriminated based on their drift times. This approach significantly enhances the separation of highly complex samples. The UPLC‐HDMS^E^ method was applied to analyze the synthetic peptide mixtures in 12 groups (Rmix1−Rmix12), each containing 20–22 Arg‐peptides, along with PAD2 or PAD4 treated mixtures where citrullinated species co‐occurred with varying prevalence and intensity. Optimal separation was achieved using an Acquity Premier Peptide HSS T3 reversed‐phase column, specifically designed to enhance the retention of small, polar peptides. The method combined a long chromatographic gradient with single‐pass cyclic ion‐mobility separation, resulting in complete retention and baseline separation of these highly polar, short, and structurally similar peptides (Figure 2A).

Arginine‐ and citrulline‐containing peptides were identified from the MS^E^ spectra. Given the high complexity and significant sequence similarity of the peptides, we opted for manual interpretation of the MS^E^ spectra to ensure reliable identification. It is important to note that several technical difficulties [22, 23, 24] hinder the accurate identification of citrullinated peptides through various data analysis software. Typically, CID fragmentation of citrullinated peptides produced a complete or nearly complete *y*‐ion series, with neutral losses of H_2_O and NH_3_ detected primarily from *y* fragment ions, but also from precursors and *b*‐ions. The neutral loss of HNCO from citrulline‐containing *y*‐ions (*y*_5–8_ marked with *y*_5_°–*y*_8_°) provided unambiguous identification of citrullination based on the MS/MS spectra of the modified peptides (Figure 2B). Additionally, through careful manual inspection of the MS^E^ spectra of Asn/Gln‐containing peptides, and the detection of specific *b‐* and *y‐*ion with high mass accuracy, we confidently ruled out the occurrence of possible deamidation. For instance, in the MS^E^ spectrum of the H*‐*ASANXKASA*‐*NH_2_ peptide, the presence of the citrulline‐containing *y*_5_ ion (*m/z* 532.615), and the asparagine‐containing *b*_4_ ion (*m/z* 344.157) were definitive markers of citrullination (Figures S1 and S2).

### 2.3 Specificity of the PAD2 and PAD4 Enzymes

The primary objective of this study was to investigate and compare the substrate specificity of PAD2 and PAD4 enzymes. Using synthetic peptides with amino acid substitutions at two distinct positions, we systematically analyzed citrullination efficiency by PAD2 and PAD4. We evaluated the impact of 16 natural amino acids in all possible combinations adjacent to the citrullination site, providing a comprehensive, multidimensional substrate preference screen. Specifically, we incorporated all amino acids directly preceding and following the central Arg residue. A total of 256 synthetic peptides were subjected to citrullination by PAD2 or PAD4, while control samples were left untreated. The efficiency of citrullination was determined by liquid chromatography‐mass spectrometry (LC‐MS) by comparing enzyme‐treated/untreated ratios.

**FIGURE 3:**
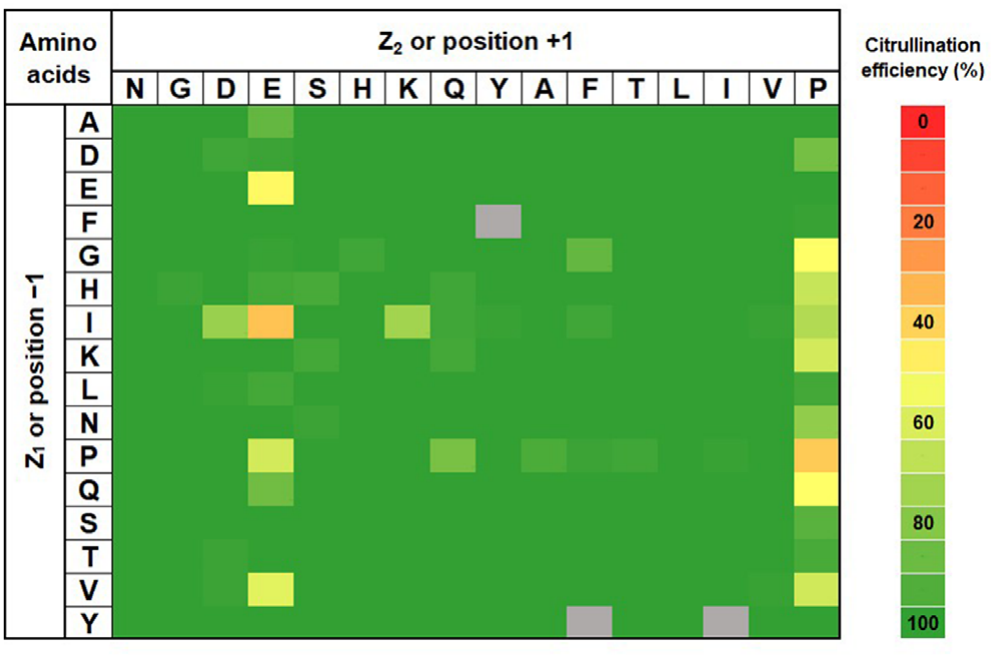
Citrullination efficiencies of H‐ASAZ_1_RZ_2_ASA‐NH_2_ peptides by PAD2. The combined influence of amino acids at the *N*‐terminal (Z_1_ or position – 1) and *C*‐terminal (Z_2_ or position + 1) sides of the target arginine is shown. Citrullination efficiency (%) is calculated using Equation (1); green indicates complete citrullination of the arginine‐containing peptides, red represents no citrullination, and grey denotes missing data. Peptide concentration: 10 μM; peptide : enzyme ratio = 20 : 1.

**FIGURE 4:**
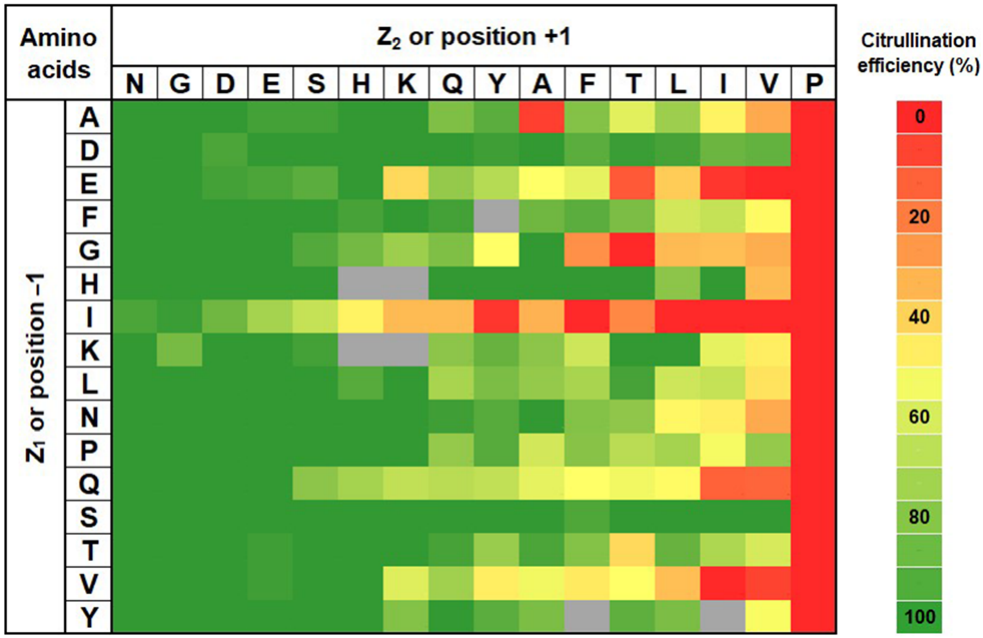
Citrullination efficiencies of H‐ASAZ_1_RZ_2_ASA‐NH_2_ peptides by PAD4. The combined influence of amino acids at the *N*‐terminal (Z_1_ or position – 1) and *C*‐terminal (Z_2_ or position + 1) sides of the target arginine is shown. Citrullination efficiency (%) is calculated using equation (**1**); green indicates complete citrullination of the arginine‐containing peptides, red represents no citrullination, and grey denotes missing data. Peptide concentration: 10 μM; peptide : enzyme ratio = 20 : 1.

Through detailed UHPLC‐HDMS^E^ analysis, we characterized the distinct roles of residues surrounding the citrullination site in the H‐ASAZ_1_RZ_2_ASA‐NH_2_ model peptide. Citrullination efficiencies for the 256 synthetic peptides by PAD2 and PAD4 are displayed as heatmaps in Figures 3 and 4. Amino acids on the N‐terminal side (position −1 or Z_1_) of the target arginine are presented vertically, while residues on the *C*‐terminal side (position +1 or Z_2_) are shown horizontally. The citrullination efficiency for each peptide is color‐coded: green represents complete citrullination, red indicates no citrullination, and grey denotes missing data. A full characterization of the peptides, including retention times, peak areas, and citrullination efficiency (conversion %) is provided in Tables S1–S12.

Our results revealed that most peptides were fully citrullinated by PAD2 regardless of sequence context, with only a few amino acid substitutions leading to reduced citrullination levels. As shown in Figure 3, two amino acids ‐ Glu and Pro ‐ at position +1 had a significant impact on citrullination efficiency, while substitutions on the *N*‐terminal side of Arg had a less pronounced effect. When glutamic acid was present at the position +1, citrullination efficiency decreased by 36%–85% in some combinations. Proline at position +1 was also largely unfavorable, except when paired with Ala, Asp, Phe, Leu, Ser, Thr, or Tyr on the *N*‐terminal side of Arg. Notably, Pro at −1 position had a less detrimental effect on PAD2‐mediated conversion. The most inhibited combinations were Pro‐Arg‐Pro and Ile‐Arg‐Glu, with efficiencies reduced to 38% and 36%, respectively. Overall, our data indicate that nearly all amino acid residues promote citrullination. In fact, 233 out of the 256 peptides were citrullinated with at least 92% efficiency. Reduced citrullination was primarily observed in combinations containing polar, acidic Glu or nonpolar Pro residues at position +1.

In a similar manner, we examined the substrate specificity of PAD4 using the same set of 256 peptides with the H‐ASAZ_1_RZ_2_ASA‐NH_2_ general sequence. Our results revealed a distinct substrate specificity profile for PAD4, with several proximity effects observed even within the −1 to +1 positional range, corroborating previously published data. As shown in Figure 4, certain amino acids on the *N*‐terminal side of Arg have a fixed influence on citrullination, while in most cases, the effect ‐ positive or negative ‐ of Z_1_ amino acids depends on the identity of the Z_2_ residue. Amino acids on the *C*‐terminal side of the Arg exhibit diverse effects on citrullination, indicating that both residues flanking the citrullination site play a unique role in enzymatic recognition and modification.

Our data show that Asp, His and Ser on the *N*‐terminal side consistently promote citrullination regardless of the amino acid at +1 position, except when Pro is present. Conversely, Ile at position −1 is unfavorable, leading to moderately or significantly reduced citrullination efficiency (64%–85% with 3 amino acids or 0%–47% with 11 amino acids). When Glu, Gln or Val precedes Arg, citrullination is reduced to varying extents (0%–78%) in more than half of the combinations.

At position +1, Asn, Gly, Asp, and Glu consistently promote citrullination. Most Ser and His substitutions at position +1 enhance citrullination, except when Ile or Gln are present at position −1, as their negative effects are more pronounced. Four amino acids – Lys, Gln, Tyr, and Ala – generally have a positive impact on citrullination, though specific Z_1_RZ_2_ combinations can significantly influence the enzyme's catalytic efficiency. In contrast, Phe, Thr, Leu, Ile, and Val are mostly unfavorable at position +1, often leading to reduced or completely inhibited citrullination. Citrullination efficiency drops by up to 50% for 7 of the 16 Z_1_ amino acids when Ile is at position +1, and for 11 of the 16 Z_1_ when Val is present. Enzymatic activity is entirely suppressed when Pro is at position +1, confirming previous findings that the Arg‐Pro sequence obstructs citrullination of the Arg residue [2, 40].

Furthermore, a clear correlation emerges between the effects of amino acids flanking the target arginine and their physicochemical properties. Notably, polar acidic, basic or neutral amino acids (with the exception of Thr) in position +1 enhance citrullination, while hydrophobic side chains in the same position are generally detrimental. Thus, it can be concluded that the charge and polarity of the amino acids directly following the arginine play a crucial role in citrullination.

## 3 Discussion

Our results reinforce and expand the current understanding of the substrate specificity of PAD2 and PAD4 enzymes. Human PAD isoforms display distinct enzymatic characteristics including variations in substrate specificities, sensitivity to physicochemical conditions, and differences in tissue and cellular distributions. Traditional approaches have characterized PAD variants based on kinetic parameters, specific protein targets, or the number of citrullinated sites detected in selected proteins. Méchin et al. reported that PAD2 catalyzed the citrullination of His‐filaggrin at least six‐times faster than other isoforms [33]. Additionally, PAD2 modified more arginine residues than PAD4 in α‐ and β‐chains of human fibrinogen [34], myelin basic protein [35] and β/γ‐actin [38]. Another study confirmed that PAD2 operates with low substrate specificity, effectively citrullinating both cellular substrates and synthetic peptides [41]. Although His, Met, Asn and Tyr were classified as disfavored residues at position −1 in cell lysate analyses, single and multiple amino acid substitutions at the peptide level still resulted in citrullination underscoring the relatively low selectivity of PAD2 [41].

Our systematic investigation further emphasizes that only in 13 out of the 256 possible amino acid combinations at positions ±1 moderately inhibit PAD2‐mediated citrullination. Moreover, only a few specific residues—namely Ile and Pro at −1, and Glu and Pro at position +1—slightly reduce the substrate potential of these peptides, in all the other 240 cases, citrullination efficiency exceeded 75%, underscoring the remarkably low substrate specificity of the PAD2 enzyme.

For PAD4, both our findings and those of others indicate significantly greater substrate specificity compared to PAD2. Early studies, based on the observed regioselective citrullinations, proposed that PAD4 activity is heavily influenced by amino acids surrounding the citrullination site [14, 34, 35, 38]. Two studies, similar to our approach, explored in detail the effects of individual amino acids flanking the central arginine. Stensland et al. systematically substituted each amino acid from −2 to +2 position in peptides derived from filaggrin and histone H3, categorizing residues as favorable or unfavorable for citrullination. Interestingly, while some amino acid preferences overlapped between histone and filaggrin, unique preferences were also identified. Notably, Ala at position +1 had a contradictory effect: it was favorable for filaggrin, but unfavorable for histone [40]. Assohou‐Luty et al. analyzed citrullination in transfected COS‐1 and HEK293 cell lysates, classifying flanking residues as preferred or disfavored based on deimination frequency in PAD‐transfected cell lysate. Further insights into PAD4 specificity were gained by screening citrullination of single amino acid substitution peptide analogs. Again, shared and distinct residue preferences emerged depending on whether cell lysates or peptide sets were analyzed [41]. The substrate specificity of PAD2 and PAD4 was also investigated by Laposchan et al. (preprint), who performed experimental proteome‐wide citrullination mapping by LC–MS/MS, identifying ~30,000 sites across ~5500 proteins (including PAD1–4), with particular focus on favored amino acid residues flanking arginines [42]. Previously reported amino acid preferences at positions flanking citrullinated arginine residues in PAD2 and PAD4 substrates are summarized in Table 1, focusing on studies with larger sample sizes.

**TABLE 1:** Amino acid preferences flanking citrullinated arginine residues in PAD2 and PAD4 substrates.

Position	PAD isoform	Favored residues	Disfavored residues	Source(s)
−1	PAD4	D, S, W	P, V	Stensland, 2009
+1	PAD4	G, N	I, V	Stensland, 2009
−1	PAD2	—	H, M, N, Y	Assohou‐Luty, 2014
+1	PAD2	G	F	Assohou‐Luty, 2014
−1	PAD4	D, G, R, S	I, K, N, Q, T	Assohou‐Luty, 2014
+1	PAD4	D, G, M	H, I, K, L, T, W	Assohou‐Luty, 2014
−1	PAD2	D, E, K, I, A	n.d.	Laposchan, 2025 (preprint)
+1	PAD2	I, K, D, V, E, L	n.d.	Laposchan, 2025 (preprint)
−1	PAD4	D, S, T	n.d.	Laposchan, 2025 (preprint)
+1	PAD4	E, D, G, N	n.d.	Laposchan, 2025 (preprint)

Several favorable amino acids, such as Asp and Ser at −1 and Gly at +1 position, identified across all experiments were confirmed by our study as generally favorable residues (Figure 4). Likewise, Ile at +1 was consistently found to be disfavored across all studies, including ours. Specific preferences, such as Thr and Tyr at position −1 (in filaggrin‐derived analogs), Asn, His and Tyr at position +1 (in histone‐derived analogs), and Asp at position +1 (in filaggrin derived analogs and cell lysates) were also validated in our results. Moreover, several disfavored residues—such as Gln at position −1, and Leu, Val, and Pro—were confirmed in a sequential context. However, some differences were observed between the Assohou‐Luty et al. [41] datasets and ours; for example, His and Lys at position +1, which were identified as disfavored in their study, were mostly favored in our analysis.

Uncertainties, such as the precise role of Ala at position +1, have been clarified through our comprehensive 2D study. We clearly demonstrated that while Ala generally promotes citrullination in many contexts, the Ala‐Arg‐Ala sequence completely blocks citrullination by PAD4. This finding provides a clear explanation for why Stensland et al. observed Ala as unfavorable at +1 position in histone, but not in filaggrin [40]. Our results show that the contradictory effect of Ala at position +1 is determined by the amino acid preceding Arg in the original histone and filaggrin sequences. Specifically, Ala‐Arg‐Ala (histone) and Gly‐Arg‐Ala (filaggrin) have opposite effects on citrullination. Through our analysis, previously contradictory or poorly understood effects of various amino acids surrounding key citrullination sites are now explained within the broader context of sequential complexity.

It is worth noting that citrullination is strongly favored by both PAD2 and PAD4 when Asn is present at either the *C*‐ or *N*‐terminal side of the target Arg. This introduces a further challenge in confidently distinguishing protein citrullination from deamidation in complex biological systems. Many citrullinated arginines in human fibrinogen [34] or those identified during PAD4 autocitrullination [43], are adjacent to an Asn residue (Arg‐Asn or Asn‐Arg). If deamidation is excluded as fixed or variable modification during citrullination analysis [44], or if stringent identification criteria are not applied, citrullination can easily be misinterpreted as deamidation. This is particularly problematic when neighboring residues are the deciding factor, especially since full sequence coverage is rarely achieved in high‐throughput proteomics.

To the best of our knowledge, this is the first systematic study incorporating amino acid substitutions at positions −1 and +1 relative to the target arginine, to provide a multidimensional substrate preference matrix. However, this study faces some inherent limitations. First, while amino acids at the immediate ±1 positions are crucial, residues further along the sequence may also play significant roles in enzyme recognition and specificity. Notably, strong interactions have been observed between PAD4 and histone proteins from positions −2 to +1 [9]. However, the complex effects of residues beyond the ±1 positions were not considered here, as systematically analyzing these would be highly challenging due to the exponential increase in peptide numbers. Furthermore, factors beyond primary sequence, such as secondary and tertiary structural effects, subcellular location, citrullination site accessibility, and neighboring PTMs can all influence deimination by PADs [40].

## 4 Conclusion

In summary, our workflow, utilizing a synthetic combinatorial peptide library offers a multidimensional, in‐depth investigation of PAD2 and PAD4 substrate specificity, paving the way for more comprehensive understanding of clinically relevant deiminated proteins. Our approach quantifies the contribution of each individual amino acid within the substrate matrix with illustrative examples, expanding the current knowledge on PAD2 and PAD4 enzymes. We underscore the critical importance of accurately distinguishing between citrullination and deamidation, especially when the post‐translationally modified amino acids are in close sequential proximity. Our results are consistent with previous structural studies of PAD2 and PAD4, which indicate greater flexibility around the active site in the case of PAD2 [45, 46, 47]. This increased flexibility may result in lower substrate specificity, which is supported by our findings.

## 5 Experimental Section

### 5.1 Synthesis

All amino acid derivatives, the Rink‐amide MBHA resin, *N*, *N′*‐diisopropyl carbodiimide (DIC), and trifluoroacetic acid (TFA) were purchased from Iris Biotech GmbH (Marktredwitz, Germany). Oxyma Pure and triisopropylsilane (TIS) were obtained from Sigma–Aldrich Kft. (Budapest, Hungary), 1,8‐diazabicyclo [5.4.0]undec‐7‐ene (DBU) was acquired from TCI Europe N.V. (Zwijndrecht, Belgium), and piperidine was purchased from Molar Chemicals Kft. (Budapest, Hungary). Human recombinant PAD2 and PAD4 enzymes were purchased from Cayman Chemical (Ann Arbor, Michigan, USA).

Peptides were manually synthesized using the combinatorial solid‐phase peptide synthesis method [48] and the Fmoc/tBu strategy, on 725 mg Rink‐amide MBHA resin (0.69 mmol/g). Elongation of the sequence was performed by sequential Fmoc removal and coupling of the corresponding amino acid. Fmoc‐protecting group was removed with 2% piperidine/2% DBU/DMF (2 + 2 + 5 +10 min). Coupling of the Fmoc amino acids (3 eq) was performed by treatment with DIC (3 eq) and Oxyma Pure (3 eq) in DMF under stirring at room temperature for 1.5 h. Before coupling the Z_2_ residue, the resin was split into 16 groups for the coupling of each Fmoc‐ and sidechain‐protected amino acid: A, D, E, F, G, H, I, K, L, N, P, Q, S, T, V, Y. Next, Fmoc‐Arg(Pbf)‐OH was coupled to each portion of resin, followed by removal of the Fmoc group. Afterwards, 12 groups (Rmix1–Rmix12) were created by mixing 11–14 different preloaded resins with the NH_2_‐RZ_2_ASA‐resin sequence in according to Tables S1–S12. For example, NH_2_‐RAASA‐resin was added to all vessels except for 5 and 9, while NH_2_‐RDASA‐resin was added to all vessels except for 3, 5, 7, and 8. Fmoc‐Z_1_‐OH amino acids were coupled based on Tables S1‐S12 (e.g., D, E, F, H, I, N, S, T were coupled to group 1; A, D, E, G, L, T, Y were coupled to group 2; etc.). Each sequence was finalized by sequentially coupling Fmoc‐Ala‐OH, Fmoc‐Ser‐(^*t*^Bu)‐OH, and Fmoc‐Ala‐OH. The resin mixtures were treated with 10 mL of cleavage solution containing 95% TFA, 2.5% H_2_O and 2.5% TIS (V/V), stirred for 2 h, filtered and washed with TFA. The filtrate was concentrated under reduced pressure, and the residue was precipitated with cold Et_2_O. The crude peptide mixtures were decantated and freeze‐dried.

### 5.2 In Vitro Citrullination Assays

Each of the 12 peptide library groups (10 μM, calculated based on the average MW of the 20–22 peptides in each group) was preincubated in 100 mM TRIS buffer (pH = 7.65) containing 10 mM CaCl_2_ and 5 mM DTT for 5 min at 37°C. For semiquantitative analysis, the peptide H‐ASAAVLASA‐NH_2_ (0.4 μM), which lacks any convertible arginine, was added as an internal standard. At the start of the experiment, PAD2 or PAD4 (0.5 μM; peptide: enzyme ratio = 20 : 1) was added and the samples were incubated for 4 h at 37°C. After incubation, the enzymatic reaction was stopped by adding formic acid, and samples were immediately frozen. Control samples without the enzyme were prepared in the same manner. Before LC‐MS analysis, the samples were vortexed, centrifuged, and transferred to glass vials.

### 5.3 LC‐HDMS^E^ Analysis

MS experiments were conducted using a high‐resolution hybrid quadrupole‐time‐of‐flight mass spectrometer (Waters Select Series Cyclic IMS, Waters Corp., Wilmslow, UK) equipped with an electrospray ionization source. Chromatographic separation was performed using a Waters Acquity I‐Class UPLC system, which was directly coupled on‐line to the mass spectrometer. The analyses were carried out in positive ion mode, with single Lock Mass calibration (leucine enkephalin, *m/z* 556.2766) employed for mass correction.

The ACQUITY PREMIER Peptide HSS T3 column (100 Å, 1.8 μm, 2.1 × 150 mm) was used for the chromatographic separation of peptides, with the column temperature maintained at 45°C. The mobile phase consisted of eluent A, 0.1% (V/V) formic acid in water, and eluent B, 0.1% (V/V) formic acid in acetonitrile. Gradient elution was carried out under the following conditions: 0 min at 0% B, 2 min at 2% B, 41 min at 20% B, 41.2 min at 80% B, 41.5 min at 80% B, 41.7 min at 0% B, and 45 min 0% B. The flow rate was set at 400 μL/min.

Data‐independent acquisition with single‐pass cyclic ion mobility (HDMS^E^) was performed using the following parameters: capillary voltage at 2.8 kV, source temperature at 120°C, and desolvation temperature at 400°C. The collision voltage was set at 4 V in full MS scan low energy mode. Ion mobility separation was performed using helium as the drift gas, with an IMS separation/cycle time of 78 ms. The Trap DC entrance was set to 0 V, and the wave height to 40 V. Data‐independent HDMS^E^ acquisition employed a collision voltage ramp from 15 to 50 V in the Transfer cell with data acquired across an *m/z* range of 50−1200.

### 5.4 Data Analysis

MS^E^ spectra of doubly charged precursors were extracted from the chromatograms using MassLynx v4.2 and visualized with mMass 5.0.0 [49]. The *b* and *y* fragment ions generated by collision‐induced dissociation from arginine‐containing and citrullinated peptides were identified and annotated in the spectra with a mass accuracy limit of 5 ppm.

Doubly protonated molecules of arginine‐containing peptides (detected within a mass window of 0.5 Da) were analyzed in the test samples subjected to enzymatic reactions and compared with control samples. Peak areas in the extracted ion chromatograms of these doubly protonated peptide ions were normalized against the peak area of an internal peptide standard, H‐ASAAVLASA‐NH_2_, which lacks arginine. This internal standard was consistently used in both the test and control samples.

Citrullination efficiency was calculated using the following formula

(1) $$\text{Citrullination efficiency}\left(\%\right)=100-\frac{{\mathrm{RPA}}_{S}}{{\mathrm{RPA}}_{\text{ctrl}}}\times 100$$

where RPA_S_ and RPA_ctrl_ represent the relative peak areas of arginine‐containing peptides in the enzyme‐treated test sample (*A*_S_) and the untreated control sample (*A*_ctrl_), respectively, both normalized to the area of the internal standard (*A*_IS_).

Experiments confirmed that the observed decrease in RPA_S_ compared to RPA_ctrl_ is solely due to citrullination, with no other processes affecting the peak area of arginine‐containing peptides in the enzyme‐treated samples.

## Funding

This study was supported by Magyar Tudományos Akadémia (Momentum Programme), Nemzeti Kutatási, Fejlesztési és Innovaciós Alap (2018−1.2.1‐NKP‐2018−00005), Innovációs és Technológiai Minisztérium (TKP2020‐NKA‐06).

## Conflicts of Interest

The authors declare no conflicts of interest.

## Supporting information

The citrullination efficiencies of *H*‐ASAZ_1_RZ_2_ASA‐*NH*_2_ peptides by PAD2 and PAD4 enzymes are summarized in Tables S1‐S12. Figures S1‐S2 presents the MS^E^ spectrum of the *H*‐ASANXKASA‐*NH*_2_ peptide.

## Data Availability

The data that support the findings of this study are available from the corresponding author upon reasonable request.
