# Supplementary material for: Mapping the Substrate Specificity Landscape of PAD2 and PAD4 Enzymes
Source: Chembiochem. 2026 Jun 7;27(11):e70408. doi: 10.1002/cbic.70408 (PMC13243971; doi:10.1002/cbic.70408)
Supplement: Supplementary file 1 — The citrullination efficiencies of H‐ASAZ1RZ2ASA‐NH 2 peptides by PAD2 and PAD4 enzymes are summarized in Tables S1‐S12. Figures S1‐S2 presents the MSE spectrum of the H‐ASANXKASA‐NH 2 peptide. [file CBIC-27-e70408-s001.pdf]

Supporting information

Mapping the Substrate Specificity Landscape of PAD2 and PAD4 Enzymes

Adina Borbély,[a] Arnold Steckel,[a] Dávid Papp, [a][b] Domonkos Pál, [c][d] Katalin Uray,[a][e] Viktória Goldschmidt Gőz,[a] and Gitta Schlosser*[a]

[a] MTA-ELTE Lendület (Momentum) Ion Mobility Mass Spectrometry Research Group and Department of Analytical Chemistry
ELTE Eötvös Loránd University, Faculty of Science, Institute of Chemistry, Department of Analytical Chemistry, Pázmány Péter sétány 1/A, H-1117 Budapest, Hungary
[b] Hevesy György PhD School of Chemistry, ELTE Eötvös Loránd University, Faculty of Science, Institute of Chemistry , Pázmány Péter sétány 1/A, H-1117, Budapest, Hungary
[c] MTA-TTK Lendület (Momentum) Glycan Biomarker Research Group, Institute of Organic Chemistry, HUN-REN Research Centre for Natural Sciences, Magyar tudósok körútja 2. H-1117, Budapest, Hungary
[d] Doctoral School, Semmelweis University, Budapest, Hungary
[e] HUN-REN–ELTE Research Group of Peptide Chemistry, ELTE Eötvös Loránd University, Faculty of Science, Institute of Chemistry, Pázmány Péter sétány 1/A, H-1117 Budapest, Hungary

*Corresponding Author

Email: gitta.schlosser@ttk.elte.hu

Tables S1-S12:

Citrullination efficiencies by PAD2 and PAD4 enzymes, calculated *m/z* of the singly and doubly protonated peptides and retention times (R_t) of the 256, arginine-containing *H*-ASAZ_1RZ_2ASA-NH_2 peptides and the citrullinated peptides (*H*-ASAZ_1XZ_2ASA-NH_2). Peak areas for the arginine-containing peptides in the enzyme-treated test sample (A_S), and in the untreated control (*Actrl*) were obtained from the extracted ion chromatograms of the doubly protonated peptides. Relative peak areas (RPA_S and RPA_{ctrl}) were determined by dividing the peak area of the sample and control groups with the peak area of the internal standard (IS) peptide *H*-ASAAVLASA-NH_2. Citrullination efficiency (%) was then calculated using Equation **1**.

**Table S1: Rmix1**				**PAD2**				**PAD4**			
Nr	Sequence	*m/z* for $[M+H]^+$	*m/z* for $[M+2H]^{2+}$	R_t (min)	A_{ctrl}	A_S	**Citr. eff. %**	R_t (min)	A_{ctrl}	A_S	**Citr. eff. %**
1	H-ASASRAASA-NH_2	790.4166	395.7119	5.39	1380	0	**100**	6.23	5626	0	**100**
1	H-ASASXAASA-NH_2	791.4006	396.2039	8.44		615		9.15		384	
2	H-ASATRSASA- NH_2	820.4272	410.7172	5.05	1924	0	**100**	5.88	6874	0	**100**
2	H-ASATXSASA- NH_2	821.4112	411.2092	7.60		176		8.30		867	
3	H-ASASRDASA- NH_2	834.4064	417.7068	5.55	2122	0	**100**	5.55	6986	0	**100**
3	H-ASASXDASA- NH_2	835.3904	418.1989	7.07		237		7.71		601	
4	H-ASADRPASA- NH_2	844.4272	422.7172	7.68	4276	680	**84**	8.46	13178	9331	**0**
4	H-ASADXPASA- NH_2	845.4112	423.2092	8.99		235		9.83		402	
5	H-ASAIRTASA- NH_2	846.4792	423.7432	11.48	3678	0	**100**	12.63	13067	6308	**22**
5	H-ASAIXTASA- NH_2	847.4632	424.2352	13.15		0		14.29		400	
6	H-ASATRNASA- NH_2	847.4381	424.2227	4.92	3585	0	**100**	5.75	11967	0	**100**
6	H-ASATXNASA- NH_2	848.4221	424.7147	7.34		515		8.00		1940	
7	H-ASAFRAASA- NH_2	850.4530	425.7301	14.67	7946	0	**100**	15.90	24513	2286	**85**
7	H-ASAFXAASA- NH_2	851.4370	426.2221	19.84		786		20.97		2092	
8	H-ASAIRIASA- NH_2	858.5156	429.7614	20.77	4936	0	**100**	22.27	13940	8650	**0**
8	H-ASAIXIASA- NH_2	859.4996	430.2534	26.12		567		24.33		743	
9	H-ASANRLASA- NH_2	859.4744	430.2409	11.88	7593	0	**100**	12.97	9947	3167	**48**
9	H-ASANXLASA- NH_2	860.4585	430.7329	16.56		654		14.57		176	
10	H-ASANRNASA- NH_2	860.4333	430.7203	3.27	3830	0	**100**	4.54	10844	0	**100**
10	H-ASANXNASA- NH_2	861.4173	431.2123	6.28		504		6.90		1347	
11	H-ASATRKASA- NH_2	861.4901	431.2487	1.87	2210	0	**100**	2.59	5297	0	**100**
11	H-ASATXKASA- NH_2	862.4741	431.7407	5.54		4075		6.31		9758	
12	H-ASAHRTASA- NH_2	870.4540	435.7307	1.78	1976	0	**100**	2.42	4753	0	**100**
12	H-ASAHXTASA- NH_2	871.4381	436.2227	5.31		3593		6.04		6330	
13	H-ASAERIASA- NH_2	874.4741	437.7407	13.03	4249	0	**100**	14.14	12726	7564	**3**
13	H-ASAEXIASA- NH_2	875.4581	438.2327	17.75		422		15.60		668	
14	H-ASADREASA- NH_2	876.4170	438.7121	6.05	4647	90	**98**	6.78	14708	0	**100**
14	H-ASADXEASA- NH_2	877.4010	439.2041	8.47		546		9.16		0	
15	H-ASAIRHASA- NH_2	882.4904	441.7489	6.85	3759	0	**100**	8.04	8639	2837	**47**
15	H-ASAIXHASA- NH_2	883.4744	442.2409	10.55		9572		11.56		13078	
16	H-ASAERKASA- NH_2	889.4850	445.2461	6.85	3759	0	**100**	3.06	4550	1664	**41**
16	H-ASAEXKASA- NH_2	890.4690	445.7381	5.82		4789		6.54		5651	
17	H-ASAFRDASA- NH_2	894.4428	447.7250	13.85	5157	0	**100**	14.97	12371	0	**100**
17	H-ASAFXDASA- NH_2	895.4268	448.2170	18.45		674		19.50		1395	
18	H-ASAFRQASA- NH_2	907.4744	454.2409	13.17	7961	0	**100**	14.27	20629	571	**96**
18	H-ASAFXQASA- NH_2	908.4585	454.7329	17.90		1705		18.91		3664	
19	H-ASADRYASA- NH_2	910.4377	455.7225	10.98	3912	0	**100**	11.90	11308	227	**97**
19	H-ASADXYASA- NH_2	911.4217	456.2145	14.59		612		15.40		1283	
20	H-ASAERYASA- NH_2	924.4534	462.7303	11.70	5819	0	**100**	12.63	13521	2676	**68**
20	H-ASAEXYASA- NH_2	925.4374	463.2223	15.74		927		16.58		1477	
21	H-ASAHRYASA- NH_2	932.4697	466.7385	6.47	2595	0	**100**	7.44	4664	0	**100**
21	H-ASAHXYASA- NH_2	933.4537	467.2305	9.87		5014		10.73		8896	
IS	H-ASAAVLASA-NH_2	759.4300	380.2216	22.48	9828	9674	**N/A**	23.82	14748	9072	**N/A**

Table S2: Rmix2				PAD2				PAD4			
Nr	Sequence	*m/z* for $[M+H]^+$	*m/z* for $[M+2H]^{2+}$	R_t (min)	A_{ctrl}	A_S	**Citr. eff. %**	R_t (min)	A_{ctrl}	A_S	**Citr. eff. %**
1	H-ASAARGASA-NH2	760.4060	380.7067	4.61	1231	0	**100**	5.60	8630	0	**100**
1	H-ASAAXGASA-NH2	761.3900	381.1987	7.34		987		8.00		1054	
2	H-ASAARAASA-NH2	774.4217	387.7145	6.18	1901	0	**100**	7.05	6774	4768	**5**
2	H-ASAAXAASA-NH2	775.4057	388.2065	9.54		1137		10.26		1512	
3	H-ASAGRTASA-NH2	790.4166	395.7119	5.00	3162	0	**100**	5.80	10832	9152	**0**
3	H-ASAGXTASA-NH2	791.4006	396.2039	7.39		929		8.04		762	
4	H-ASAGRDASA-NH2	804.3959	402.7016	4.59	2142	0	**100**	5.67	5062	0	**100**
4	H-ASAGXDASA-NH2	805.3799	403.1936	6.87		467		7.50		714	
5	H-ASAARNASA-NH2	817.4275	409.2174	4.46	2693	0	**100**	5.50	5500	0	**100**
5	H-ASAAXNASA-NH2	818.4115	409.7094	7.2		546		7.80		3644	
6	H-ASAGRHASA-NH2	826.4278	413.7176	1.54	1443	0	**100**	2.17	4153	478	**84**
6	H-ASAGXHASA-NH2	827.4118	414.2096	4.37		3561		5.44		17786	
7	H-ASAAREASA-NH2	832.4272	416.7172	6.28	3807	447	**88**	7.09	10947	332	**96**
7	H-ASAAXEASA-NH2	833.4112	417.2092	9.29		1954		10.04		1964	
8	H-ASATRTASA-NH2	834.4428	417.7250	5.86	4033	0	**100**	6.60	11016	4786	**41**
8	H-ASATXTASA-NH2	835.4268	418.2170	8.56		435		9.28		1436	
9	H-ASATRLASA-NH2	846.4792	423.7432	13.14	7831	0	**100**	14.30	18920	1866	**87**
9	H-ASATXLASA-NH2	847.4632	424.2352	18.17		804		19.16		2320	
10	H-ASAARFASA-NH2	850.4530	425.7301	15.14	6481	0	**100**	16.31	16488	2486	**80**
10	H-ASAAXFASA-NH2	851.4370	426.2221	20.36		623		21.39		1606	
11	H-ASALRIASA-NH2	858.5156	429.7614	21.72	13557	0	**100**	23.20	32048	8514	**64**
11	H-ASALXIASA-NH2	859.4996	430.2534	27.48		1362		28.85		3454	
12	H-ASADRIASA-NH2	860.4585	430.7329	12.71	8022	0	**100**	13.70	18316	1918	**86**
12	H-ASADXIASA-NH2	861.4425	431.2249	16.83		836		17.75		2014	
13	H-ASADRDASA-NH2	862.4013	431.7043	5.26	2331	70	**97**	6.85	5789	250	**94**
13	H-ASADXDASA-NH2	863.3854	432.1963	7.38		196		7.55		506	
14	H-ASATRHASA-NH2	870.4540	435.7307	1.98	2316	0	**100**	6.24	3979	0	**100**
14	H-ASATXHASA-NH2	871.4381	436.2227	5.69		5359		6.46		21996	
15	H-ASAERLASA-NH2	874.4741	437.7407	13.61	10171	0	**100**	14.68	21493	9896	**38**
15	H-ASAEXLASA-NH2	875.4581	438.2327	18.56		1358		19.49		1480	
16	H-ASAERDASA-NH2	876.4170	438.7121	5.84	3311	0	**100**	6.35	8225	222	**96**
16	H-ASAEXDASA-NH2	877.4010	439.2041	8.96				8.96		2068	
17	H-ASALRHASA-NH2	882.4904	441.7489	7.49	3976	0	**100**	9.40	7932	558	**91**
17	H-ASALXHASA-NH2	883.4744	442.2409	11.54		11937		12.59		18006	
18	H-ASAYRVASA-NH2	894.4792	447.7432	14.88	5062	0	**100**	15.97	9908	3594	**51**
18	H-ASAYXVASA-NH2	895.4632	448.2352	19.45		724		20.42		928	
19	H-ASAYRDASA-NH2	910.4377	455.7225	9.78	4405	0	**100**	10.59	1138	0	**100**
19	H-ASAYXDASA-NH2	911.4217	456.2145	13.35		625		14.10		0	
20	H-ASAYRQASA-NH2	923.4694	462.2383	9.44	3196	0	**100**	10.24	11701	0	**100**
20	H-ASAYXQASA-NH2	924.4534	462.7303	13.13		713		13.86		1264	
IS	H-ASAAVLASA-NH2	759.4300	380.2216	22.47	10406	10528	**N/A**	23.73	11075	8222	**N/A**

**Table S3: Rmix3**				**PAD2**				**PAD4**			
Nr	Sequence	*m/z* for $[M+H]^+$	*m/z* for $[M+2H]^{2+}$	R_t (min)	A_{ctrl}	A_S	**Citr. eff. %**	R_t (min)	A_{ctrl}	A_S	**Citr. eff. %**
1	H-ASAGRAASA-NH2	760.4060	380.7067	5.21	1483	0	**100**	5.98	3644	0	**100**
1	H-ASAGXAASA-NH2	761.3900	381.1987	7.93		1011		8.59		472	
2	H-ASAARPASA-NH2	800.4373	400.7223	7.63	1817	0	**100**	8.39	3102	5613	**0**
2	H-ASAAXPASA-NH2	801.4213	401.2143	10.59		493		ND		0	
3	H-ASADRGASA-NH2	804.3959	402.7016	4.67	1319	0	**100**	5.55	2502	0	**100**
3	H-ASADXGASA-NH2	805.3799	403.1936	6.99		239		7.60		554	
4	H-ASAARTASA-NH2	804.4322	402.7198	5.75	2352	0	**100**	6.57	4369	2225	**57**
4	H-ASAAXTASA-NH2	805.4163	403.2118	8.60		794		9.29		350	
5	H-ASAGRQASA-NH2	817.4275	409.2174	4.29	2418	0	**100**	5.39	3680	842	**81**
5	H-ASAGXQASA-NH2	818.4115	409.7094	6.91		220		7.54		670	
6	H-ASAHRGASA-NH2	826.4278	413.7176	1.54	6600	0	**100**	1.73	1959	0	**100**
6	H-ASAHXGASA-NH2	827.4118	414.2096	3.89		3422		5.16		4374	
7	H-ASAERAASA-NH2	832.4272	416.7172	6.64	3073	0	**100**	7.42	4535	2733	**50**
7	H-ASAEXAASA-NH2	833.4112	417.2092	9.86		245		10.55		611	
8	H-ASAFRGASA-NH2	836.4373	418.7223	13.06	6501	0	**100**	14.13	8774	0	**100**
8	H-ASAFXGASA-NH2	837.4213	419.2143	17.47		615		18.45		2138	
9	H-ASAIRVASA-NH2	844.4999	422.7536	16.94	5143	37	**99**	18.24	7434	11678	**0**
9	H-ASAIXVASA-NH2	845.4839	423.2456	21.82		513		20.21		632	
10	H-ASAVRNASA-NH2	845.4588	423.2330	7.49	3892	0	**100**	8.36	5557	0	**100**
10	H-ASAVXNASA-NH2	846.4428	423.7250	10.71		522		11.47		472	
11	H-ASAIRLASA-NH2	858.5156	429.7614	21.31	8653	0	**100**	22.75	11732	14967	**0**
11	H-ASAIXLASA-NH2	859.4996	430.2534	26.86		1016		24.85		722	
12	H-ASADRLASA-NH2	860.4585	430.7329	13.23	7167	0	**100**	14.24	8656	485	**95**
12	H-ASADXLASA-NH2	861.4425	431.2249	17.62		769		18.49		2186	
13	H-ASAERTASA-NH2	862.4377	431.7225	6.19	3470	0	**100**	6.92	4408	4672	**11**
13	H-ASAEXTASA-NH2	863.4217	432.2145	8.90		462		9.55		782	
14	H-ASAHRPASA-NH2	866.4591	433.7332	5.25	1615	420	**64**	6.12	1748	2826	**0**
14	H-ASAHXPASA-NH2	867.4431	434.2252	8.08		2855		8.82		1085	
15	H-ASAIRQASA-NH2	873.4901	437.2487	10.68	7796	1531	**73**	11.67	9745	7647	**34**
15	H-ASAIXQASA-NH2	874.4741	437.7407	14.99		1134		15.85		2642	
16	H-ASADRKASA-NH2	875.4694	438.2383	2.09	1802	0	**100**	2.80	1559	0	**100**
16	H-ASADXKASA-NH2	876.4534	438.7303	5.34		4340		5.99		7829	
17	H-ASAHRNASA-NH2	883.4493	442.2283	1.54	3600	3500	**99**	1.66	1721	0	**100**
17	H-ASAHXNASA-NH2	884.4333	442.7203	3.70		3389		5.00		2973	
18	H-ASAEREASA-NH2	890.4326	445.7200	6.66	4284	1578	**49**	7.38	4841	332	**94**
18	H-ASAEXEASA-NH2	891.4167	446.2120	9.57		387		10.23		2204	
19	H-ASAVRYASA-NH2	894.4792	447.7432	14.44	5970	0	**100**	15.50	5620	3792	**43**
19	H-ASAVXYASA-NH2	895.4632	448.2352	18.95		792		19.98		1418	
20	H-ASAFRKASA-NH2	907.5108	454.2591	8.78	3242	0	**100**	9.95	3233	0	**100**
20	H-ASAFXKASA-NH2	908.4948	454.7511	13.25		9660		14.27		17142	
21	H-ASAFRHASA-NH2	916.4748	458.7410	9.04	3086	0	**100**	10.25	3687	222	**95**
21	H-ASAFXHASA-NH2	917.4588	459.2330	13.36		10268		14.40		20240	
IS	H-ASAAVLASA-NH2	759.4300	380.2216	22.46	10726	10498	**N/A**	23.70	6492	7748	**N/A**

Table S4: Rmix4				PAD2				PAD4			
Nr	Sequence	*m/z* for [M+H]$^+$	*m/z* for [M+2H]$^{2+}$	R_t (min)	A_{ctrl}	A_S	**Citr. eff. %**	R_t (min)	A_{ctrl}	A_S	**Citr. eff. %**
1	H-ASAPRAASA-NH2	800.4373	400.7223	8.94	4074	229	**94**	9.83	11726	3251	**61**
1	H-ASAPXAASA-NH2	801.4213	401.2143	2.04		179		11.27		0	
2	H-ASATRAASA-NH2	804.4322	402.7198	6.24	2420	0	**100**	7.18	6777	289	**94**
2	H-ASATXAASA-NH2	805.4163	403.2118	9.48		167		10.31		486	
3	H-ASAGRKASA-NH2	817.4639	409.2356	1.56	2040	0	**100**	1.92	4537	851	**74**
3	H-ASAGXKASA-NH2	818.4479	409.7276	3.83		3571		5.25		6334	
4	H-ASAPRPASA-NH2	826.4530	413.7301	10.56	3292	2060	**38**	11.49	7627	5549	**0**
4	H-ASAPXPASA-NH2	827.4370	414.2221	11.96		352		ND		0	
5	H-ASAIRSASA-NH2	832.4635	416.7354	10.17	4486	0	**100**	11.23	10742	2771	**64**
5	H-ASAIXSASA-NH2	833.4476	417.2274	14.33		378		12.93		0	
6	H-ASAGRFASA-NH2	836.4373	418.7223	13.53	7034	892	**88**	14.68	16614	9003	**24**
6	H-ASAGXFASA-NH2	837.4213	419.2143	15.29		477		16.62		532	
7	H-ASALRVASA-NH2	844.4999	422.7536	17.73	8113	0	**100**	19.29	17364	7013	**43**
7	H-ASALXVASA-NH2	845.4839	423.2456	23.09		379		21.21		467	
8	H-ASAQRSASA-NH2	847.4381	424.2227	4.32	3266	0	**100**	5.50	7958	1243	**78**
8	H-ASAQXSASA-NH2	848.4221	424.7147	7.07		424		7.70		515	
9	H-ASAYRGASA-NH2	852.4322	426.7198	9.11	6215	0	**100**	10.00	11085	0	**100**
9	H-ASAYXGASA-NH2	853.4163	427.2118	12.5		913		13.41		1584	
10	H-ASAQRPASA-NH2	857.4588	429.2330	7.59	3266	1652	**50**	8.42	6987	5866	**0**
10	H-ASAQXPASA-NH2	858.4428	429.7250	8.86		277		9.11		228	
11	H-ASALRLASA-NH2	858.5156	429.7614	22.37	13696	0	**100**	24.09	24738	6622	**62**
11	H-ASALXLASA-NH2	859.4996	430.2534	28.35		3750		29.90		2888	
12	H-ASAIRDASA-NH2	860.4585	430.7329	11.18	5241	1342	**75**	12.28	11533	1261	**85**
12	H-ASAIXDASA-NH2	861.4425	431.2249	14.03		48		16.44		1231	
13	H-ASATREASA-NH2	862.4377	431.7225	6.33	3685	0	**100**	7.22	7798	156	**97**
13	H-ASATXEASA-NH2	863.4217	432.2145	7.47		52		10.09		1026	
14	H-ASALRQASA-NH2	873.4901	437.2487	11.41	8091	0	**100**	12.62	15334	3186	**71**
14	H-ASALXQASA-NH2	874.4741	437.7407	16.04		1373		17.17		2462	
15	H-ASAPRFASA-NH2	876.4686	438.7380	18.32	9486	230	**98**	19.67	19978	2947	**79**
15	H-ASAPXFASA-NH2	877.4526	439.2300	22.41		860		23.65		1576	
16	H-ASAKREASA-NH2	889.4850	445.2461	2.04	2536	0	**100**	2.97	3744	0	**100**
16	H-ASAKXEASA-NH2	890.4690	445.7381	4.62		386		6.53		6466	
17	H-ASAPRYASA-NH2	892.4635	446.7354	13.36	5327	0	**100**	14.37	10910	717	**91**
17	H-ASAPXYASA-NH2	893.4476	447.2274	16.66		538		17.63		1003	
18	H-ASATRYASA-NH2	896.4585	448.7329	11.35	6178	0	**100**	12.43	11416	2006	**75**
18	H-ASATXYASA-NH2	897.4425	449.2249	15.55		720		16.56		0	
19	H-ASAQRFASA-NH2	907.4744	454.2409	13.83	8693	0	**100**	15.05	15385	5484	**50**
19	H-ASAQXFASA-NH2	908.4585	454.7329	18.77		596		16.76		301	
20	H-ASAKRFASA-NH2	907.5108	454.2591	9.37	4818	0	**100**	10.74	4621	1240	**62**
20	H-ASAKXFASA-NH2	908.4948	454.7511	14.17		7607		15.34		7484	
21	H-ASAYRYASA-NH2	958.4741	479.7407	15.71	5483	0	**100**	16.76	8390	575	**90**
21	H-ASAYXYASA-NH2	959.4581	480.2327	20.34		1001		21.36		1358	
IS	H-ASAAVLASA-NH_2	759.4300	380.2216	22.47	10695	10872	**N/A**	23.92	11087	7890	**N/A**

Table S5: Rmix5				PAD2				PAD4			
Nr	Sequence	*m/z* for [M+H]$^{+}$	*m/z* for [M+2H]$^{2+}$	R_t (min)	A_{ctrl}	A_S	**Citr. eff. %**	R_t (min)	A_{ctrl}	A_S	**Citr. eff. %**
1	H-ASASRGASA-NH2	776.4009	388.7041	3.36	1649	0	**100**	4.59	2092	0	**100**
1	H-ASASXGASA-NH2	777.3850	389.1961	6.49		602		7.15		260	
2	H-ASAARVASA-NH2	802.4530	401.7301	9.68	3929	0	**100**	10.71	5072	2420	**30**
2	H-ASAAXVASA-NH2	803.4370	402.2221	13.89		290		12.09		230	
3	H-ASAKRGASA-NH2	817.4639	409.2356	1.28	1723	0	**100**	1.64	1338	152	**83**
3	H-ASAKXGASA-NH2	818.4479	409.7276	3.53		2458		4.76		2449	
4	H-ASAVRPASA-NH2	828.4686	414.7380	10.25	3303	1709	**62**	11.18	3783	3205	**0**
4	H-ASAVXPASA-NH2	829.4526	415.2300	11.84		420		0.00		0	
5	H-ASALRSASA-NH2	832.4635	416.7354	10.82	6236	0	**100**	11.90	6489	0	**100**
5	H-ASALXSASA-NH2	833.4476	417.2274	15.35		568		16.72		839	
6	H-ASAARHASA-NH2	840.4435	420.7254	1.79	1923	0	**100**	2.80	1172	0	**100**
6	H-ASAAXHASA-NH2	841.4275	421.2174	5.44		4258		6.24		3432	
7	H-ASAVRIASA-NH2	844.4999	422.7536	17.08	8637	0	**100**	18.37	8425	5736	**0**
7	H-ASAVXIASA-NH2	845.4839	423.2456	22.04		2843		20.32		540	
8	H-ASASRQASA-NH2	847.4381	424.2227	4.29	3099	0	**100**	5.39	3212	0	**100**
8	H-ASASXQASA-NH2	848.4221	424.7147	7.04		675		7.62		588	
9	H-ASASRKASA-NH2	847.4744	424.2409	1.48	2134	0	**100**	1.99	1508	0	**100**
9	H-ASASXKASA-NH2	848.4585	424.7329	4.62		3666		5.54		2447	
10	H-ASAKRVASA-NH2	859.5108	430.2591	5.47	2921	0	**100**	6.98	2110	778	**46**
10	H-ASAKXVASA-NH2	860.4948	430.7511	9.11		5274		9.96		2706	
11	H-ASAERVASA-NH2	860.4585	430.7329	10.08	5008	0	**100**	11.05	4143	3084	**0**
11	H-ASAEXVASA-NH2	861.4425	431.2249	14.13		601		12.35		238	
12	H-ASAARYASA-NH2	866.4479	433.7276	11.29	4226	0	**100**	12.22	3805	265	**90**
12	H-ASAAXYASA-NH2	867.4319	434.2196	15.53		262		16.32		0	
13	H-ASAQRIASA-NH2	873.4901	437.2487	11.69	8769	0	**100**	12.71	7385	4368	**13**
13	H-ASAQXIASA-NH2	874.4741	437.7407	16.33		971		14.12		510	
14	H-ASALRKASA-NH2	873.5265	437.2669	7.49	4129	0	**100**	8.95	2553	0	**100**
14	H-ASALXKASA-NH2	874.5105	437.7589	11.71		10053		10.26		120	
15	H-ASAFRVASA-NH2	878.4843	439.7458	20.25	10268	28	**100**	21.63	8819	3047	**49**
15	H-ASAFXVASA-NH2	879.4683	440.2378	25.68		1270		23.71		255	
16	H-ASAKRKASA-NH2	888.5374	444.7723	1.10	2480	135	**96**	0.00		0	**ND**
16	H-ASAKXKASA-NH2	889.5214	445.2643	1.54		3726		2.03		797	
17	H-ASAYRPASA-NH2	892.4635	446.7354	12.03	2865	0	**100**	12.90	2483	1883	**0**
17	H-ASAYXPASA-NH2	893.4476	447.2274	16.12		427		14.57		0	
18	H-ASAYRTASA-NH2	896.4585	448.7329	10.09	5511	0	**100**	10.95	4534	339	**89**
18	H-ASAYXTASA-NH2	897.4425	449.2249	13.82		796		14.57		654	
19	H-ASAERFASA-NH2	908.4585	454.7329	15.25	7082	0	**100**	16.33	5227	1575	**56**
19	H-ASAEXFASA-NH2	909.4425	455.2249	20.25		1164		21.16		607	
20	H-ASAQRYASA-NH2	923.4694	462.2383	10.57	7134	0	**100**	11.42	4982	1185	**65**
20	H-ASAQXYASA-NH2	924.4534	462.7303	14.49		886		15.22		533	
21	H-ASAFRFASA-NH2	926.4843	463.7458	27.09	13935	69	**100**	28.58	9268	647	**90**
21	H-ASAFXFASA-NH2	927.4686	464.2378	33.05		2326		34.34		1608	
IS	H-ASAAVLASA-NH_2	759.4300	380.2216	22.47	11060	14974	**N/A**	23.64	12114	8228	**N/A**

Table S6: Rmix6				PAD2				PAD4			
Nr	Sequence	*m/z* for $[M+H]^+$	*m/z* for $[M+2H]^{2+}$	R_t (min)	A_{ctrl}	A_S	**Citr. eff. %**	R_t (min)	A_{ctrl}	A_S	**Citr. eff. %**
1	H-ASAPRGASA-NH2	786.4217	393.7145	7.89	2312	0	**100**	8.62	5340	0	**100**
1	H-ASAPXGASA-NH2	787.4057	394.2065	10.13		77		10.77		266	
2	H-ASAPRSASA-NH2	816.4322	408.7198	7.81	3319	0	**100**	8.53	6049	0	**100**
2	H-ASAPXSASA-NH2	817.4163	409.2118	10.00		145		10.64		441	
3	H-ASAARLASA-NH2	816.4686	408.7380	13.26	6026	0	**100**	14.42	11957	2205	**74**
3	H-ASAAXLASA-NH2	817.4526	409.2300	18.44		491		19.33		1101	
4	H-ASAARDASA-NH2	818.4115	409.7094	5.44	3234	0	**100**	6.22	6340	0	**100**
4	H-ASAAXDASA-NH2	819.3955	410.2014	8.06		188		8.69		613	
5	H-ASAVRSASA-NH2	818.4479	409.7276	7.71	3294	0	**100**	8.62	7232	0	**100**
5	H-ASAVXSASA-NH2	819.4319	410.2196	11.10		229		11.87		605	
6	H-ASASRTASA-NH2	820.4272	410.7172	5.04	2695	0	**100**	5.81	5094	0	**100**
6	H-ASASXTASA-NH2	821.4112	411.2092	7.63		261		8.23		600	
7	H-ASAPRVASA-NH2	828.4686	414.7380	13.09	7225	23	**100**	14.07	14412	4495	**57**
7	H-ASAPXVASA-NH2	829.4526	415.2300	16.23		384		0.00		0	
8	H-ASASRIASA-NH2	832.4635	416.7354	11.41	4862	0	**100**	12.43	8779	0	**100**
8	H-ASASXIASA-NH2	833.4476	417.2274	16.08		439		16.90		1099	
9	H-ASAHRAASA-NH2	840.4435	420.7254	1.71	2187	0	**100**	2.62	2531	0	**100**
9	H-ASAHXAASA-NH2	841.4275	421.2174	5.46		2898		6.18		4832	
10	H-ASAVRLASA-NH2	844.4999	422.7536	17.58	10248	0	**100**	18.87	18174	8534	**35**
10	H-ASAVXLASA-NH2	845.4839	423.2456	22.79		996		23.87		1176	
11	H-ASALRTASA-NH2	846.4792	423.7432	12.05	8863	0	**100**	13.15	15302	528	**95**
11	H-ASALXTASA-NH2	847.4632	424.2352	16.63		900		17.56		2031	
12	H-ASASREASA-NH2	848.4221	424.7147	5.43	3168	0	**100**	6.18	5179	0	**100**
12	H-ASASXEASA-NH2	849.4061	425.2067	8.08		330		8.68		718	
13	H-ASASRHASA-NH2	856.4384	428.7228	1.56	2241	0	**100**	2.12	2504	0	**100**
13	H-ASASXHASA-NH2	857.4224	429.2148	4.95		3565		5.67		6341	
14	H-ASALRNASA-NH2	859.4744	430.2409	10.61	7600	0	**100**	11.66	13116	0	**100**
14	H-ASALXNASA-NH2	860.4585	430.7329	14.88		1130		15.74		2331	
15	H-ASAFRSASA-NH2	866.4479	433.7276	12.63	6154	0	**100**	13.69	9439	0	**100**
15	H-ASAFXSASA-NH2	867.4319	434.2196	17.32		613		18.24		1175	
16	H-ASAFRPASA-NH2	876.4686	438.7380	15.98	8377	98	**99**	17.09	12457	9975	**0**
16	H-ASAFXPASA-NH2	877.4526	439.2300	31.88		47		22.12		318	
17	H-ASAVRFASA-NH2	878.4843	439.7458	19.63	11015	19	**100**	20.90	17030	6787	**45**
17	H-ASAVXFASA-NH2	879.4683	440.2378	24.89		1263		25.99		1318	
18	H-ASAHRDASA-NH2	884.4333	442.7203	1.77	1765	0	**100**	2.36	2034	0	**100**
18	H-ASAHXDASA-NH2	885.4173	443.2123	4.91		2813		5.61		4657	
19	H-ASAFRIASA-NH2	892.4999	446.7536	24.32	15404	0	**100**	25.77	21037	5388	**64**
19	H-ASAFXIASA-NH2	893.4839	447.2456	30.10		1827		31.33		2376	
20	H-ASAFRNASA-NH2	893.4588	447.2330	12.49	6578	0	**100**	13.53	8714	0	**100**
20	H-ASAFXNASA-NH2	894.4428	447.7250	16.98		1160		17.88		2005	
21	H-ASAHRQASA-NH2	897.4649	449.2361	1.54	875	0	**100**	2.03	2047	0	**100**
21	H-ASAHXQASA-NH2	898.4490	449.7281	4.73		3498		5.54		3746	
22	H-ASAFREASA-NH2	908.4585	454.7329	14.61	9462	0	**100**	15.68	12346	0	**100**
22	H-ASAFXEASA-NH2	909.4425	455.2249	19.53		1254		20.46		2065	
IS	H-ASAAVLASA-NH_2	759.4300	380.2216	22.47	10868	10315	**N/A**	23.61	11821	8510	**N/A**

Table S7: Rmix7				PAD2				PAD4			
Nr	Sequence	*m/z* for [M+H]$^+$	*m/z* for [M+2H]$^{2+}$	R_t (min)	A_{ctrl}	A_S	**Citr. eff. %**	R_t (min)	A_{ctrl}	A_S	**Citr. eff. %**
1	H-ASAGRPASA-NH2	786.4217	393.7145	6.92	2399	1192	**50**	7.63	12487	10232	**0**
1	H-ASAGXPASA-NH2	787.4057	394.2065	8.74		156		8.90		326	
2	H-ASAGRLASA-NH2	802.453	401.7301	11.67	5290	0	**100**	12.67	22868	10374	**34**
2	H-ASAGXLASA-NH2	803.437	402.2221	16.19		340		17.04		1183	
3	H-ASASRPASA-NH2	816.4322	408.7198	7.17	2283	207	**91**	7.85	15287	11996	**0**
3	H-ASASXPASA-NH2	817.4163	409.2118	9.91		181		10.55		168	
4	H-ASADRAASA-NH2	818.4115	409.7094	6.11	2550	0	**100**	6.88	12404	0	**100**
4	H-ASADXAASA-NH2	819.3955	410.2014	8.83		196		8.77		144	
5	H-ASAPRTASA-NH2	830.4479	415.7276	8.62	4232	140	**97**	9.37	16363	3732	**67**
5	H-ASAPXTASA-NH2	831.4319	416.2196	10.95		290		11.62		1670	
6	H-ASASRLASA-NH2	832.4635	416.7354	11.96	5715	0	**100**	12.96	22136	0	**100**
6	H-ASASXLASA-NH2	833.4476	417.2274	16.88		673		17.72		3244	
7	H-ASAIRPASA-NH2	842.4843	421.7458	12.77	4117	1280	**69**	13.79	16069	15004	**0**
7	H-ASAIXPASA-NH2	843.4683	422.2378	14.58		257		15.63		516	
8	H-ASANRVASA-NH2	845.4588	423.2330	8.53	5078	0	**100**	9.37	19485	9466	**30**
8	H-ASANXVASA-NH2	846.4428	423.7250	12.26		402		12.98		1141	
9	H-ASAKRSASA-NH2	847.4744	424.2409	1.79	6135	0	**100**	1.83	5174	133	**96**
9	H-ASAKXSASA-NH2	848.4585	424.7329	4.07		3519		5.21		11997	
10	H-ASAPRQASA-NH2	857.4588	429.2330	8.19	4633	0	**100**	8.90	17891	2936	**76**
10	H-ASAPXQASA-NH2	858.4428	429.7250	10.48		604		11.09		2276	
11	H-ASANRIASA-NH2	859.4744	430.2409	11.31	7715	0	**100**	12.26	27461	10319	**46**
11	H-ASANXIASA-NH2	860.4585	430.7329	15.72		643		16.53		1785	
12	H-ASASRFASA-NH2	866.4479	433.7276	13.66	5456	0	**100**	14.67	18131	871	**93**
12	H-ASASXFASA-NH2	867.4319	434.2196	18.74		681		19.60		2644	
13	H-ASAIRKASA-NH2	873.5265	437.2669	6.92	3709	111	**97**	8.30	9528	4372	**34**
13	H-ASAIXKASA-NH2	874.5105	437.7589	10.77		10433		11.73		18466	
14	H-ASAIREASA-NH2	874.4741	437.7407	11.98	7529	4787	**36**	13.03	22478	4408	**72**
14	H-ASAIXEASA-NH2	875.4581	438.2327	13.40		857		17.43		3647	
15	H-ASADRQASA-NH2	875.4330	438.2201	5.18	1641	0	**100**	5.86	5766	0	**100**
15	H-ASADXQASA-NH2	876.4170	438.7121	7.42		311		7.97		1166	
16	H-ASAFRTASA-NH2	880.4635	440.7354	14.20	8324	0	**100**	15.31	26597	3266	**82**
16	H-ASAFXTASA-NH2	881.4476	441.2274	18.90		1216		19.84		3841	
17	H-ASANRHASA-NH2	883.4493	442.2283	1.57	2026	41	**98**	2.04	5390	0	**100**
17	H-ASANXHASA-NH2	884.4333	442.7203	4.84		5693		5.59		17747	
18	H-ASAQRQASA-NH2	888.4646	444.7359	4.88	4485	0	**100**	5.61	15572	3597	**67**
18	H-ASAQXQASA-NH2	889.4486	445.2279	7.38		985		7.92		2706	
19	H-ASAFRLASA-NH2	892.4999	446.7536	25.04	9730	0	**100**	26.46	44869	11988	**61**
19	H-ASAFXLASA-NH2	893.4839	447.2456	30.94		2400		32.17		6662	
20	H-ASAQRHASA-NH2	897.46490	449.2361	1.65	2046	0	**100**	2.33	4785	917	**72**
20	H-ASAQXHASA-NH2	898.4490	449.7281	5.13		6364		5.80		13962	
21	H-ASAIRYASA-NH2	908.4948	454.7511	17.47	8733	65	**99**	18.56	25001	16734	**3**
21	H-ASAIXYASA-NH2	909.4789	455.2431	22.42		1371		23.35		1610	
22	H-ASAKRYASA-NH2	923.5057	462.2565	6.82	2763	0	**100**	7.83	18333	1618	**87**
22	H-ASAKXYASA-NH2	924.4898	462.7485	10.46		7283		11.24		15844	
IS	H-ASAAVLASA-NH_2	759.4300	380.2216	22.47	10870	10777	**N/A**	23.60	12184	8423	**N/A**

Table S8: Rmix8				PAD2				PAD4			
Nr	Sequence	*m/z* for $[M+H]^{+}$	*m/z* for $[M+2H]^{2+}$	R_t (min)	A_{ctrl}	A_S	**Citr. eff. %**	R_t (min)	A_{ctrl}	A_S	**Citr. eff. %**
1	H-ASAIRGASA-NH2	802.4530	401.7301	10.38	4879	0	**100**	11.32	8355	141	**98**
1	H-ASAIXGASA-NH2	803.4370	402.2221	14.21		310		15.04		630	
2	H-ASAIRAASA-NH2	816.4686	408.7380	11.95	6923	0	**100**	13.05	9449	4571	**32**
2	H-ASAIXAASA-NH2	817.4526	409.2300	16.62		548		17.50		531	
3	H-ASAERGASA-NH2	818.4115	409.7094	5.16	3391	0	**100**	5.86	4963	0	**100**
3	H-ASAEXGASA-NH2	819.3955	410.2014	7.62		281		8.19		490	
4	H-ASATRPASA-NH2	830.4479	415.7276	7.91	2136	135	**95**	8.64	3924	3060	**0**
4	H-ASATXPASA-NH2	831.4319	416.2196	9.25		154				0	
5	H-ASATRVASA-NH2	832.4635	416.7354	9.62	4764	0	**100**	10.56	7458	2138	**60**
5	H-ASATXVASA-NH2	833.4476	417.2274	13.72		414		12.04		129	
6	H-ASALRPASA-NH2	842.4843	421.7458	13.53	4248	196	**96**	14.59	7744	6278	**0**
6	H-ASALXPASA-NH2	843.4683	422.2378	15.38		214		16.48		229	
7	H-ASAHRSASA-NH2	856.4384	428.7228	1.44	1733	0	**100**	1.85	1726	0	**100**
7	H-ASAHXSASA-NH2	857.4224	429.2148	4.24		2783		5.30		3469	
8	H-ASAIRNASA-NH2	859.4744	430.2409	9.91	5954	0	**100**	10.84	9512	410	**94**
8	H-ASAIXNASA-NH2	860.4585	430.7329	13.86		907		14.64		0	
9	H-ASADRNASA-NH2	861.4173	431.2123	4.54	2917	0	**100**	5.41	4661	0	**100**
9	H-ASADXNASA-NH2	862.4013	431.7043	6.79		493		7.34		912	
10	H-ASATRQASA-NH2	861.4537	431.2305	5.48	4290	0	**100**	6.22	5943	234	**95**
10	H-ASATXQASA-NH2	862.4377	431.7225	8.05		696		8.62		1000	
11	H-ASAYRAASA-NH2	866.4479	433.7276	10.29	5484	0	**100**	11.14	6429	849	**82**
11	H-ASAYXAASA-NH2	867.4319	434.2196	14.41		627		15.13		670	
12	H-ASAKRIASA-NH2	873.5265	437.2669	7.55	4759	0	**100**	9.07	4854	1517	**56**
12	H-ASAKXIASA-NH2	874.5105	437.7589	11.90		9568		12.77		8065	
13	H-ASALREASA-NH2	874.4741	437.7407	12.81	10351	533	**96**	13.86	12239	0	**100**
13	H-ASALXEASA-NH2	875.4581	438.2327	17.72		1212		18.61		1887	
14	H-ASAERNASA-NH2	875.4330	438.2201	5.05	3733	0	**100**	5.75	5022	0	**100**
14	H-ASAEXNASA-NH2	876.4170	438.7121	7.42		647		7.98		935	
15	H-ASADRHASA-NH2	884.4333	442.7203	2.37	1794	0	**100**	3.31	2455	0	**100**
15	H-ASADXHASA-NH2	885.4173	443.2123	5.60		4466		6.24		5109	
16	H-ASAKRQASA-NH2	888.5010	444.7541	1.49	492	0	**100**	1.99	1843	296	**78**
16	H-ASAKXQASA-NH2	889.4850	445.2461	4.62		3371		5.48		3790	
17	H-ASAIRFASA-NH2	892.4999	446.7536	23.35	13902	595	**97**	24.67	14160	10534	**0**
17	H-ASAIXFASA-NH2	893.4839	447.2456	28.88		1820		26.76		643	
18	H-ASAHRKASA-NH2	897.5013	449.2543	1.11	2759	101	**97**	ND		0	**ND**
18	H-ASAHXKASA-NH2	898.4853	449.7463	1.56		4871		2.07		1582	
19	H-ASALRYASA-NH2	908.4948	454.7511	18.81	10869	0	**100**	19.93	11312	1444	**82**
19	H-ASALXYASA-NH2	909.4789	455.2431	24.20		1574		25.15		1597	
20	H-ASAHRFASA-NH2	916.4748	458.7410	9.25	5211	0	**100**	10.59	2404	0	**100**
20	H-ASAHXFASA-NH2	917.4588	459.2330	13.69		6257		14.62		6635	
21	H-ASAYRKASA-NH2	923.5057	462.2565	6.11	2844	0	**100**	7.17	2810	402	**80**
21	H-ASAYXKASA-NH2	924.4898	462.7485	9.62		7395		10.37		6137	
IS	H-ASAAVLASA-NH_2	759.4300	380.2216	22.47	12130	15201	**N/A**	23.58	10968	7857	**N/A**

Table S9: Rmix9				PAD2				PAD4			
Nr	Sequence	*m/z* for [M+H]$^+$	*m/z* for [M+2H]$^{2+}$	R_t (min)	A_{ctrl}	A_S	**Citr. eff. %**	R_t (min)	A_{ctrl}	A_S	**Citr. eff. %**
1	H-ASAGRSASA-NH2	776.4009	388.7041	3.68	2203	69	**97**	4.96	4793	280	**92**
1	H-ASAGXSASA-NH2	777.3850	389.1961	6.49		138		7.07		421	
2	H-ASAGRVASA-NH2	788.4373	394.7223	8.40	4024	13	**100**	9.27	8741	4658	**31**
2	H-ASAGXVASA-NH2	798.4213	395.2143	11.98		178		10.65		0	
3	H-ASALRGASA-NH2	802.4530	401.7301	10.97	6126	0	**100**	11.95	11931	0	**100**
3	H-ASALXGASA-NH2	803.4370	402.2221	15.15		388		15.98		1048	
4	H-ASAGREASA-NH2	818.4115	409.7094	5.42	4525	77	**99**	6.12	6983	0	**100**
4	H-ASAGXEASA-NH2	819.3955	410.2014	7.83		225		8.40		647	
5	H-ASAVRVASA-NH2	830.4843	415.7458	13.57	7126	61	**99**	14.71	12454	9058	**6**
5	H-ASAVXVASA-NH2	831.4683	416.2378	17.98		521		18.91		411	
6	H-ASAVRTASA-NH2	832.4635	416.7354	8.82	4876	26	**100**	9.75	9089	3542	**50**
6	H-ASAVXTASA-NH2	833.4476	417.2274	12.33		445		13.10		762	
7	H-ASAPRIASA-NH2	842.4843	421.7458	16.19	12788	165	**99**	17.22	21708	8069	**52**
7	H-ASAPXIASA-NH2	843.4683	422.2378	19.84		809		20.73		1275	
8	H-ASADRVASA-NH2	846.4428	423.7250	9.68	4943	0	**100**	10.53	6739	635	**88**
8	H-ASADXVASA-NH2	847.4268	424.2170	13.23		439		13.94		863	
9	H-ASADRTASA-NH2	848.4221	424.7147	5.72	4799	0	**100**	6.39	7807	147	**98**
9	H-ASADXTASA-NH2	849.4061	425.2067	8.05		644		8.61		1160	
10	H-ASAKRPASA-NH2	857.4952	429.2512	5.21	2498	1219	**61**	6.05	2921	2305	**0**
10	H-ASAKXPASA-NH2	858.4792	429.7432	7.93		2191		ND		0	
11	H-ASAQRVASA-NH2	859.4744	430.2409	8.95	6194	0	**100**	9.82	9507	6338	**14**
11	H-ASAQXVASA-NH2	860.4585	430.7329	12.90		540		11.08		258	
12	H-ASANRDASA-NH2	861.4173	431.2123	4.92	2880	0	**100**	5.27	4878	0	**100**
12	H-ASANXDASA-NH2	862.4013	431.7043	6.82		234		7.34		449	
13	H-ASAPRHASA-NH2	866.4591	433.7332	6.03	2108	0	**100**	6.37	2344	0	**100**
13	H-ASAPXHASA-NH2	867.4431	434.2252	7.71		4980		8.36		7472	
14	H-ASAKRLASA-NH2	873.5265	437.2669	7.97	4531	0	**100**	9.42	4409	0	**100**
14	H-ASAKXLASA-NH2	874.5105	437.7589	12.62		8654		13.49		9598	
15	H-ASAKRNASA-NH2	874.4853	437.7463	1.31	2663	0	**100**	1.62	2870	0	**100**
15	H-ASAKXNASA-NH2	875.4694	438.2383	3.40		4489		4.53		6111	
16	H-ASANREASA-NH2	875.4330	438.2201	5.30	5356	0	**100**	5.96	8030	0	**100**
16	H-ASANXEASA-NH2	876.4170	438.7121	7.81		525		8.38		959	
17	H-ASAYRSASA-NH2	882.4428	441.7250	8.95	4844	0	**100**	9.71	6404	0	**100**
17	H-ASAYXSASA-NH2	883.4268	442.2170	12.58		530		13.23		973	
18	H-ASAQRKASA-NH2	888.5010	444.7541	1.61	3253	0	**100**	2.16	2958	790	**65**
18	H-ASAQXKASA-NH2	889.4850	445.2461	5.05		5750		5.71		6200	
19	H-ASALRFASA-NH2	892.4999	446.7536	24.46	16535	0	**100**	25.82	21385	4719	**71**
19	H-ASALXFASA-NH2	893.4839	447.2456	30.44		1931		31.59		2936	
20	H-ASAKRHASA-NH2	897.5013	449.2543	1.11	2715	136	**96**	ND			**ND**
20	H-ASAKXHASA-NH2	898.4853	449.7463	1.58		3737		2.17		2322	
IS	H-ASAAVLASA-NH_2	759.4300	380.2216	22.48	12680	15772	**N/A**	23.57	11101	8577	**N/A**

Table S10: Rmix10				PAD2				PAD4			
Nr	Sequence	*m/z* for [M+H]$^+$	*m/z* for [M+2H]$^{2+}$	R_t (min)	A_{ctrl}	A_S	**Citr. eff. %**	R_t (min)	A_{ctrl}	A_S	**Citr. eff. %**
1	H-ASAVRGASA-NH2	788.4373	394.7223	7.85	3174	0	**100**	8.69	8738	0	**100**
1	H-ASAVXGASA-NH2	789.4213	395.2143	11.03		153		11.74		605	
2	H-ASAVRAASA-NH2	802.4530	401.7301	9.17	3838	0	**100**	10.16	11733	3939	**53**
2	H-ASAVXAASA-NH2	803.4370	402.2221	13.13		265		13.90		631	
3	H-ASAARIASA-NH2	816.4686	408.7380	12.71	5297	0	**100**	13.81	12302	4696	**47**
3	H-ASAAXIASA-NH2	817.4526	409.2300	17.62		418		18.44		783	
4	H-ASAQRGASA-NH2	817.4275	409.2174	3.89	2801	0	**100**	5.10	6274	0	**100**
4	H-ASAQXGASA-NH2	818.4115	409.7094	6.85		206		7.36		554	
5	H-ASASRVASA-NH2	818.4479	409.7276	8.62	3718	0	**100**	9.49	7465	0	**100**
5	H-ASASXVASA-NH2	819.4319	410.2196	12.95		319		13.28		932	
6	H-ASAQRAASA-NH2	831.4431	416.2252	6.54	3430	0	**100**	6.54	6676	2093	**56**
6	H-ASAQXAASA-NH2	832.4272	416.7172	8.87		205		8.96		132	
7	H-ASAARKASA-NH2	831.4795	416.2434	1.78	2626	21	**99**	2.63	3801	0	**100**
7	H-ASAAXKASA-NH2	832.4635	416.7354	5.43		4534		6.15		6971	
8	H-ASANRSASA-NH2	833.4224	417.2148	3.56	3125	0	**100**	4.74	6021	0	**100**
8	H-ASANXSASA-NH2	834.4064	417.7068	6.58		746		7.08		616	
9	H-ASAPRLASA-NH2	842.4843	421.7458	16.48	10175	29	**100**	17.49	18330	3619	**72**
9	H-ASAPXLASA-NH2	843.4683	422.2378	20.30		738		21.19		1648	
10	H-ASAPRDASA-NH2	844.4272	422.7172	8.21	2409	0	**100**	8.87	4862	0	**100**
10	H-ASAPXDASA-NH2	845.4112	423.2092	10.42		174		10.99		394	
11	H-ASAVRDASA-NH2	846.4428	423.7250	8.49	3241	68	**98**	9.33	7667	0	**100**
11	H-ASAVXDASA-NH2	847.4268	424.2170	11.94		292		11.57		209	
12	H-ASAERSASA-NH2	848.4221	424.7147	5.31	2975	0	**100**	5.92	5516	393	**90**
12	H-ASAEXSASA-NH2	849.4061	425.2067	7.83		327		8.38		680	
13	H-ASAPRKASA-NH2	857.4952	429.2512	5.33	2300	375	**83**	6.10	3515	0	**100**
13	H-ASAPXKASA-NH2	858.4792	429.7432	7.46		4441		8.10		7699	
14	H-ASAVRQASA-NH2	859.4744	430.2409	8.22	4725	0	**100**	9.11	10562	2071	**73**
14	H-ASAVXQASA-NH2	860.4585	430.7329	11.74		719		12.45		1343	
15	H-ASAQRTASA-NH2	861.4537	431.2305	5.46	3830	0	**100**	6.15	6894	2335	**53**
15	H-ASAQXTASA-NH2	862.4377	431.7225	8.06		445		8.61		627	
16	H-ASANRKASA-NH2	874.4853	437.7463	9.42	7806	0	**100**	1.87	3222	0	**100**
16	H-ASANXKASA-NH2	875.4694	438.2383	4.31		4990		5.28		8008	
17	H-ASAQRDASA-NH2	875.4330	438.2201	5.04	2383	0	**100**	25.00	4122	0	**100**
17	H-ASAQXDASA-NH2	876.4170	438.7121	7.42		282		7.93		626	
18	H-ASASRYASA-NH2	882.4428	441.7250	10.30	4204	0	**100**	12.45	7888	0	**100**
18	H-ASASXYASA-NH2	883.4268	442.2170	14.24		515		14.90		1020	
19	H-ASAERHASA-NH2	898.4490	449.7281	2.25	2126	0	**100**	3.44	3583	0	**100**
19	H-ASAEXHASA-NH2	899.4330	450.2201	5.86		4877		6.50		6321	
20	H-ASAYRLASA-NH2	908.4948	454.7511	18.90	11060	0	**100**	19.98	15533	2000	**82**
20	H-ASAYXLASA-NH2	909.4789	455.2431	24.12		1760		25.00		2221	
21	H-ASAYREASA-NH2	924.4534	462.7303	10.46	5338	0	**100**	11.26	7542	0	**100**
21	H-ASAYXEASA-NH2	925.4374	463.2223	14.41		851		15.07		1436	
IS	H-ASAAVLASA-NH_2	759.4300	380.2216	22.47	10611	10071	**N/A**	23.53	10190	7302	**N/A**

Table S11: Rmix11				PAD2				PAD4			
Nr	Sequence	*m/z* for [M+H]$^{+}$	*m/z* for [M+2H]$^{2+}$	R_t (min)	A_{ctrl}	A_S	**Citr. eff. %**	R_t (min)	A_{ctrl}	A_S	**Citr. eff. %**
1	H-ASAGRGASA-NH2	746.3904	373.6988	3.43	1364	0	**100**	4.67	5273	0	**100**
1	H-ASAGXGASA-NH2	747.3744	374.1908	6.44		58		6.97		328	
2	H-ASAARSASA-NH2	790.4166	395.7119	4.87	2320	0	**100**	5.69	5792	143	**96**
2	H-ASAAXSASA-NH2	791.4006	396.2039	7.55		119		8.14		480	
3	H-ASAGRIASA-NH2	802.4530	401.7301	11.16	4659	0	**100**	12.10	12018	5317	**35**
3	H-ASAGXIASA-NH2	803.4370	402.2221	15.41		254		13.68		260	
4	H-ASAGRNASA-NH2	803.4118	402.2096	3.48	1769	0	**100**	4.60	5583	0	**100**
4	H-ASAGXNASA-NH2	804.3959	402.7016	6.36		175		6.90		569	
5	H-ASASRSASA-NH2	806.4115	403.7094	3.70	2295	0	**100**	4.90	4529	0	**100**
5	H-ASASXSASA-NH2	807.3955	404.2014	6.71		186		7.25		558	
6	H-ASALRAASA-NH2	816.4686	408.7380	12.79	6104	0	**100**	13.98	14171	2362	**76**
6	H-ASALXAASA-NH2	817.4526	409.2300	17.88		503		18.78		1334	
7	H-ASAARQASA-NH2	831.4431	416.2252	5.35	2806	0	**100**	6.12	6461	820	**81**
7	H-ASAAXQASA-NH2	832.4272	416.7172	8.07		410		8.64		873	
8	H-ASASRNASA-NH2	833.4224	417.2148	3.48	2728	0	**100**	4.60	5485	0	**100**
8	H-ASASXNASA-NH2	834.4064	417.7068	6.44		389		9.13		0	
9	H-ASANRPASA-NH2	843.4431	422.2252	6.99	2694	585	**77**	7.69	7116	5970	**0**
9	H-ASANXPASA-NH2	844.4272	422.7172	8.25		133		17.26		1107	
10	H-ASATRIASA-NH2	846.4792	423.7432	12.56	6499	0	**100**	13.62	13388	2761	**70**
10	H-ASATXIASA-NH2	847.4632	424.2352	17.33		685		18.17		1374	
11	H-ASANRTASA-NH2	847.4381	424.2227	4.97	3919	0	**100**	5.69	7770	1219	**77**
11	H-ASANXTASA-NH2	848.4221	424.7147	7.49		347		8.05		743	
12	H-ASAGRYASA-NH2	852.4322	426.7198	9.96	4049	0	**100**	10.76	7540	2551	**50**
12	H-ASAGXYASA-NH2	853.4163	427.2118	13.55		334		14.20		529	
13	H-ASAERPASA-NH2	858.4428	429.7250	8.23	3384	0	**100**	8.91	6737	5957	**0**
13	H-ASAEXPASA-NH2	859.4268	430.2170	11.14		349		10.27		213	
14	H-ASALRDASA-NH2	860.4585	430.7329	11.94	6758	74	**99**	12.73	11848	0	**100**
14	H-ASALXDASA-NH2	861.4425	431.2249	16.43		715		17.26		2135	
15	H-ASAHRVASA-NH2	868.4748	434.7410	5.31	2302	0	**100**	6.98	3436	1560	**34**
15	H-ASAHXVASA-NH2	869.4588	435.2330	8.62		4434		9.42		4830	
16	H-ASANRQASA-NH2	874.4490	437.7281	3.98	4287	0	**100**	5.12	8065	184	**97**
16	H-ASANXQASA-NH2	875.4330	438.2201	6.82		746		7.32		1465	
17	H-ASATRFASA-NH2	880.4635	440.7354	15.05	7030	0	**100**	16.15	12859	1781	**80**
17	H-ASATXFASA-NH2	881.4476	441.2274	20.23		939		21.11		1672	
18	H-ASAHRIASA-NH2	882.4904	441.7489	7.38	3271	0	**100**	8.90	4403	0	**100**
18	H-ASAHXIASA-NH2	883.4744	442.2409	11.29		7388		12.16		7340	
19	H-ASAERQASA-NH2	889.4486	445.2279	5.69	3400	0	**100**	6.38	5888	967	**76**
19	H-ASAEXQASA-NH2	890.4326	445.7200	8.28		748		8.87		1110	
20	H-ASANRFASA-NH2	893.4588	447.2330	13.65	7710	0	**100**	14.60	13661	1862	**80**
20	H-ASANXFASA-NH2	894.4428	447.7250	18.48		881		19.28		1452	
21	H-ASAHREASA-NH2	898.4490	449.7281	2.04	2149	83	**96**	4.46	2807	0	**100**
21	H-ASAHXEASA-NH2	899.4330	450.2201	5.56		4361		6.23		6955	
22	H-ASANRYASA-NH2	909.4537	455.2305	10.00	2231	0	**100**	10.76	8753	530	**91**
22	H-ASANXYASA-NH2	910.4377	455.7225	13.73		653		14.38		1065	
IS	H-ASAAVLASA-NH_2	759.4300	380.2216	22.47	10931	10463	**N/A**	23.53	9826	6712	**N/A**

**Table S12: Rmix12**				**PAD2**				**PAD4**			
Nr	Sequence	*m/z* for $[M+H]^+$	*m/z* for $[M+2H]^{2+}$	R_t (min)	A_{ctrl}	A_S	**Citr. eff. %**	R_t (min)	A_{ctrl}	A_S	**Citr. eff. %**
1	H-ASATRGASA-NH2	790.4166	395.7119	5.09	1790	0	**100**	5.80	4501	0	**100**
1	H-ASATXGASA-NH2	791.4006	396.2039	7.64		105		8.18		418	
2	H-ASANRGASA-NH2	803.4118	402.2096	3.30	2222	0	**100**	4.38	5097	0	**100**
2	H-ASANXGASA-NH2	804.3959	402.7016	6.43		127		6.95		473	
3	H-ASANRAASA-NH2	817.4275	409.2174	5.29	2745	0	**100**	6.00	5119	0	**100**
3	H-ASANXAASA-NH2	818.4115	409.7094	8.15		165		8.69		433	
4	H-ASAKRAASA-NH2	831.4795	416.2434	1.86	2022	0	**100**	3.46	2849	447	**78**
4	H-ASAKXAASA-NH2	832.4635	416.7354	5.92		2668		6.61		4891	
5	H-ASADRSASA-NH2	834.4064	417.7068	4.87	1515	0	**100**	6.22	3892	0	**100**
5	H-ASADXSASA-NH2	835.3904	418.1989	7.09		287		7.66		770	
6	H-ASAPRNASA-NH2	843.4431	422.2252	7.58	3324	0	**100**	8.55	7104	0	**100**
6	H-ASAPXNASA-NH2	844.4272	422.7172	9.74		338		10.29		889	
7	H-ASATRDASA-NH2	848.4221	424.7147	5.61	2554	71	**98**	6.34	5754	0	**100**
7	H-ASATXDASA-NH2	849.4061	425.2067	8.13		203		8.69		560	
8	H-ASAPREASA-NH2	858.4428	429.7250	9.07	4920	2405	**61**	9.75	9295	0	**100**
8	H-ASAPXEASA-NH2	859.4268	430.2170	11.48		216		12.09		1120	
9	H-ASAVRKASA-NH2	859.5108	430.2591	4.99	2702	0	**100**	6.60	4059	1187	**58**
9	H-ASAVXKASA-NH2	860.4948	430.7511	8.23		6102		9.12		9096	
10	H-ASAVREASA-NH2	860.4585	430.7329	9.32	5188	2798	**57**	8.55	9002	187	**97**
10	H-ASAVXEASA-NH2	861.4425	431.2249	13.14		219		13.23		1231	
11	H-ASAKRTASA-NH2	861.4901	431.2487	1.75	1728	0	**100**	2.45	2473	0	**100**
11	H-ASAKXTASA-NH2	862.4741	431.7407	5.34		3103		6.00		4701	
12	H-ASAVRHASA-NH2	868.4748	434.7410	4.92	2068	0	**100**	6.60	3113	0	**100**
12	H-ASAVXHASA-NH2	869.4588	435.2330	8.09		5249		8.90		8542	
13	H-ASAQRLASA-NH2	873.4901	437.2487	12.24	7522	0	**100**	13.20	13204	4710	**49**
13	H-ASAQXLASA-NH2	874.4741	437.7407	17.15		829		17.87		1131	
14	H-ASAQRNASA-NH2	874.4490	437.7281	3.81	3657	0	**100**	5.00	5757	0	**100**
14	H-ASAQXNASA-NH2	875.4330	438.2201	6.70		652		7.17		1314	
15	H-ASAKRDASA-NH2	875.4694	438.2383	1.75	1668	0	**100**	2.29	2497	0	**100**
15	H-ASAKXDASA-NH2	876.4534	438.7303	5.39		391		5.54		5055	
16	H-ASAHRLASA-NH2	882.4904	441.7489	7.68	2794	0	**100**	9.57	3524	550	**78**
16	H-ASAHXLASA-NH2	883.4744	442.2409	11.86		6131		12.73		8857	
17	H-ASAQREASA-NH2	889.4486	445.2279	5.86	4353	794	**85**	6.54	6705	0	**100**
17	H-ASAQXEASA-NH2	890.4326	445.7200	8.60		548		9.12		1235	
18	H-ASADRFASA-NH2	894.4428	447.7250	14.92	4625	0	**100**	15.84	10157	689	**90**
18	H-ASADXFASA-NH2	895.4268	448.2170	19.58		658		20.36		1155	
19	H-ASAHRHASA-NH2	906.4653	453.7363	1.13	1685	112	**95**	ND			**ND**
19	H-ASAHXHASA-NH2	907.4493	454.2283	1.64		1812		2.28		2008	
20	H-ASAYRNASA-NH2	909.4537	455.2305	8.87	3909	0	**100**	9.59	6621	0	**100**
20	H-ASAYXNASA-NH2	910.4377	455.7225	12.33		785		12.92		1428	
21	H-ASAYRHASA-NH2	932.4697	466.7385	6.27	1873	0	**100**	7.60	2374	0	**100**
21	H-ASAYXHASA-NH2	933.4537	467.2305	9.70		5650		10.42		7847	
IS	H-ASAAVLASA-NH_2	759.4300	380.2216	22.49	12847	16060	**N/A**	23.49	9195	6475	**N/A**

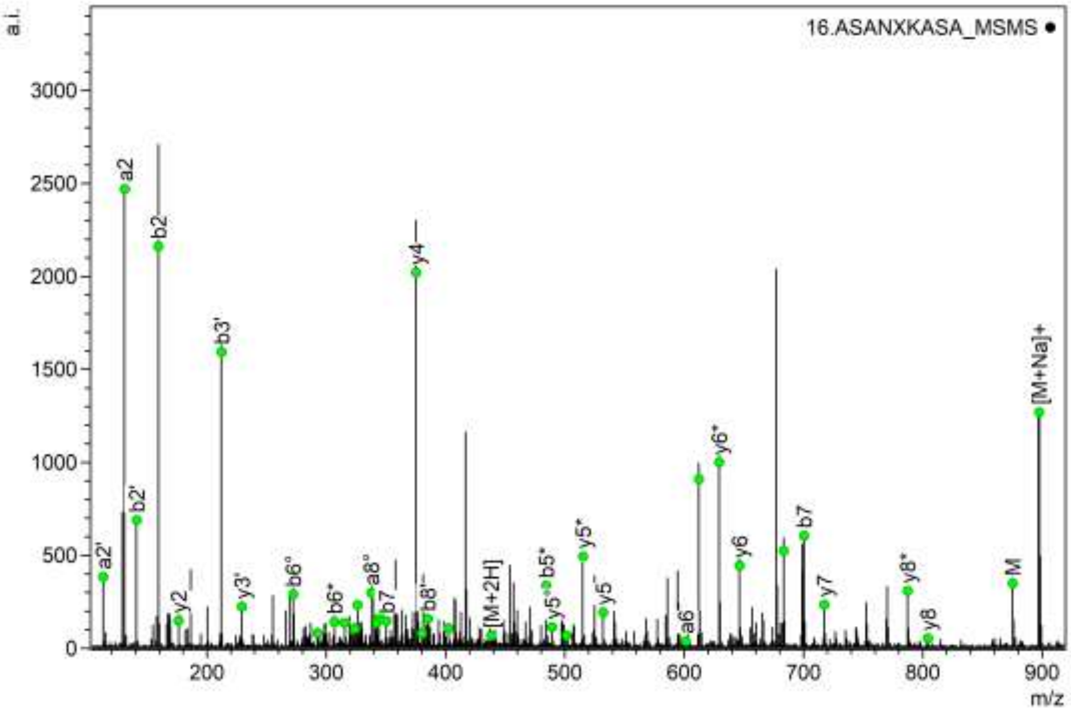

Figure S1. MS/MS spectrum of the *H*-ASANXKASA-*NH₂* peptide. Neutral losses are indicated as follows: ‘= H_2O, * = NH_3, ^{o} = HNCO. a_6, y_6 and y_8 fragments around *m/z* 300 are doubly charged fragments, all other fragments are singly charged.

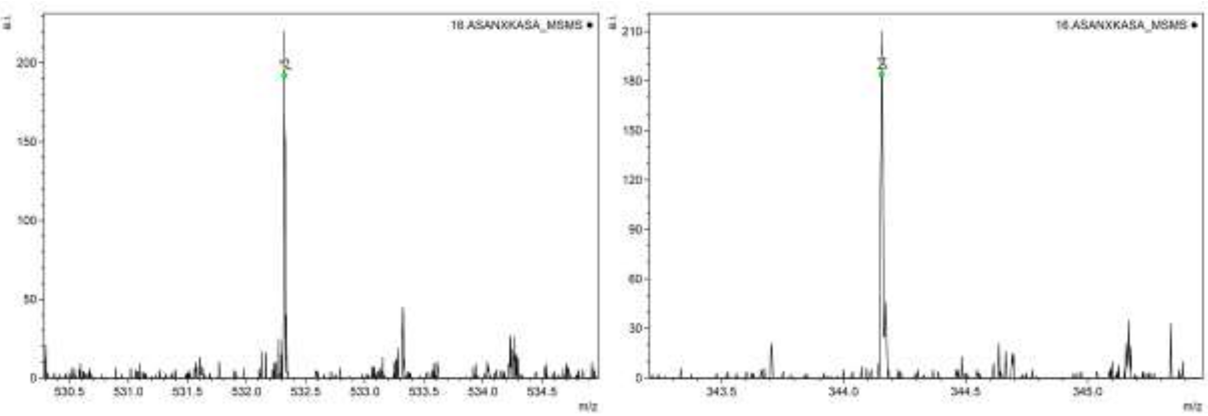

Figure S2. Enlargements of the MS/MS spectrum of the *H*-ASANXKASA-*NH₂* peptide showing the y_5 ion at *m/z* 532.6149, and the b_4 ion at *m/z* 344.3439.
